# Recent Developments of Carboxymethyl Cellulose

**DOI:** 10.3390/polym13081345

**Published:** 2021-04-20

**Authors:** Md. Saifur Rahman, Md. Saif Hasan, Ashis Sutradhar Nitai, Sunghyun Nam, Aneek Krishna Karmakar, Md. Shameem Ahsan, Muhammad J. A. Shiddiky, Mohammad Boshir Ahmed

**Affiliations:** 1 Department of Chemistry and Biochemistry, The University of Texas at El Paso, El Paso, TX 79968, USA; 2 Department of Applied Chemistry and Chemical Engineering, University of Rajshahi, Rajshahi 6205, Bangladesh; mshasan.ru@yahoo.com (M.S.H.); sd.ashis.ru5107@gmail.com (A.S.N.); aneek@ru.ac.bd (A.K.K.); shameem@ru.ac.bd (M.S.A.); 3 United States Department of Agriculture, Agricultural Research Service, Southern Regional Research Center, 1100 Robert E. Lee Boulevard, New Orleans, LA 70124, USA; sunghyun.nam@usda.gov; 4 School of Environment and Science (ESC) and Queensland Micro- and Nanotechnology Centre (QMNC), Griffith University, Nathan 4111, Australia; m.shiddiky@griffith.edu.au; 5 School of Materials Science and Engineering, Gwangju Institute of Science and Technology, Gwangju 61005, Korea

**Keywords:** CMC, wound dressing, 3D bio-printing, bio-sensing, pharmaceutical, drug delivery, water treatment, textile, food, energy production

## Abstract

Carboxymethyl cellulose (CMC) is one of the most promising cellulose derivatives. Due to its characteristic surface properties, mechanical strength, tunable hydrophilicity, viscous properties, availability and abundance of raw materials, low-cost synthesis process, and likewise many contrasting aspects, it is now widely used in various advanced application fields, for example, food, paper, textile, and pharmaceutical industries, biomedical engineering, wastewater treatment, energy production, and storage energy production, and storage and so on. Many research articles have been reported on CMC, depending on their sources and application fields. Thus, a comprehensive and well-organized review is in great demand that can provide an up-to-date and in-depth review on CMC. Herein, this review aims to provide compact information of the synthesis to the advanced applications of this material in various fields. Finally, this article covers the insights of future CMC research that could guide researchers working in this prominent field.

## 1. Introduction

**Figure 1:**
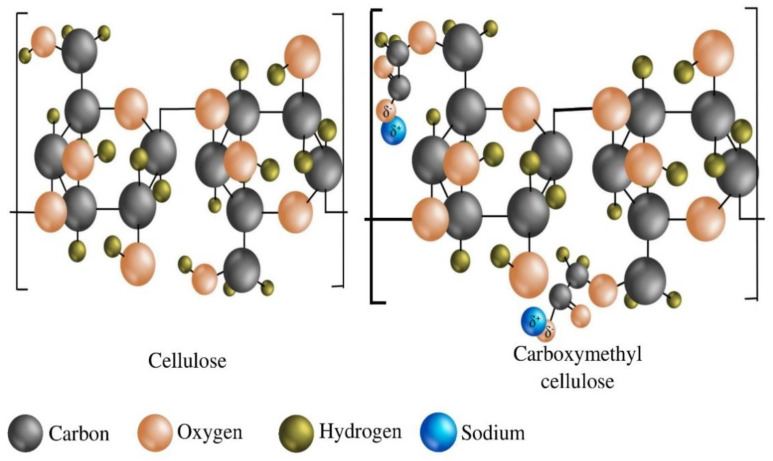
The fundamental structural difference between cellulose and carboxymethyl cellulose (CMC).

Carboxymethyl cellulose (CMC) is an anionic, water-soluble derivative of cellulose, a linear polysaccharide of anhydro-glucose. The repeating units are connected by β-1,4-glycosidic bonds. At the molecular level, the major difference between CMC and cellulose is only some anionic carboxymethyl groups (i.e., –CH_2_COOH) in the CMC structure that replace the hydrogen atoms from some hydroxyl groups present in the pristine cellulose infrastructure (Figure 1). CMC was first synthesized in 1918. However, the commercial production of these all-important polymeric materials was first depicted in Germany in the early 1920s [1].

Initially, as the archetypal celluloses were mainly extracted from wood and other plant-based precursors that contained a high percentage of cellulose fibers naturally, the production of CMC was then ultimately dependent on such specific wood-based plants [2,3,4]. However, day-by-day cellulose-containing materials have been introduced by many researchers in the literature as effectual alternative candidates in this context. Among them, some plant-based precursors (e.g., sago palm [5], corn cobs [6], corn husk [7], corn stalks [8], durian rind [9], banana pseudo-stem [10], maize stalks [11], cacao pod husks [12], the pulp of *Eucalyptus globulus* [13], orange peel [14], pineapple peel [15], sugarcane bagasse [16], *Asparagus officinalis* stalk end [17], etc.), as well as some waste-materials (such as waste paper sludge [18], wastepaper [19], knitted rags [20], waste textiles [21], cotton gin wastes [22], and waste cotton linters from textile industries [23], etc.) have attracted the attention of researchers to be applied for the bulk or commercial production of CMC.

Due to the facile, low-cost synthesis process, an abundance of raw materials, characteristic surface properties [24,25], mechanical strength [26], different formability [27,28], tuneable hydrophilicity [29,30,31,32], a viscosity [33], rheological properties [34], and likewise hundreds of other contrasting aspects, CMC and CMC-based hybrid materials have found a wide range of applications in the biomedical, pharmaceutical, textile, construction, food, plastics, cosmetics, paper, and oil industries. For instance, in biomedical fields, CMC and its composites are widely used in tissue engineering [35,36,37,38], bone-tissue engineering [39,40,41], wound dressing [31,32,42,43,44], absorbent nonwovens [30], fabrication of 3D-scaffolds for biocompatible implants [45,46], artificial organs or mimics of extracellular polymeric matrix [47,48], diagnosis of various diseases [49,50], etc. for various purposes. Over the last few years, CMC-based hydrogels, films, or other hybrid materials have gained much interest in pharmaceutical applications, especially for drug delivery [51,52], drug emulsification [53,54], and stabilization purposes [55,56] due to their excellent biocompatibility, high stability, pH sensitivity, and binding capacity against pharmaceutically active compounds like drugs, enzymes. Moreover, CMC and its derivatives are used in textile digital printing due to their thickening and color sharpening feature [57,58], in textile weaving as sizing and finishing agents [59,60], and used in smart, antimicrobial, antiradical, antioxidant, or absorbent textiles because of their pH or thermosensitivity, hygienic, and hygroscopic features [30,31,32,61,62,63]. CMCs are used as various auxiliary agents like thickeners, emulsion stabilizers, adhesive stabilizers, and moisture binders in numerous food products and their packaging due to the odorless, tasteless, non-caloric, and physiologically inert properties [26,64,65,66,67,68]. Additionally, in environmentally favorable energy production, storage, and wastewater treatment, the role of CMC in various aspects has been demonstrated by many researchers thus far in the literature. As a cost-efficient binder in biomass pellets (for reduction of excessive fuel-loss) [69,70], auxiliary material for electrodes in battery cells [71,72,73], supercapacitor aerogels for efficient energy storage [74,75], CMC and its various composites have attracted huge attention during recent years. In water treatment processes, CMC-based hybrid composites, especially their hydrogels, have demonstrated some promising results over the past two decades for effective removal of dye pollutants [76,77,78], various inorganic metal ions (including heavy metals in their complex ionic states) [79,80,81], and even some radionuclides [82,83] from various contaminated waters. Moreover, applications of CMC have also been proposed in various fields such as in the paper industry to provide strengthening property, good printability or ink retention, color stability, fire retardancy features of CMC [84,85,86,87]; in paper or wood adhesives for a better adhesion property [88,89]; and in Si anodic electrodes for their potential binding capacity and cyclic performance [90]. Additionally, CMC has been used in the construction industry [91,92], cosmetics [93], dental profession [94], liquid detergent [95], fertilizer industry [96,97], oil industry [98,99], etc.

However, the application of CMC in these fields highly depends upon the purity, degree of polymerization (DP), degree of substitution (DS), and uniformity, which govern the performance of the resulting products such as solubility, particle size, viscosity, rheological properties, etc. TypicallyCMC products are divided into three different grades: food-grade, pharmaceutical grade, and industrial grade CMC based on purity and DS [7,69]. The industrial-grade CMCs cannot be used for pharmaceutical or biomedical purposes such as drug delivery, tissue or organ implantation in vivo, wound dressing, etc., due to their impure chemical compositions and characteristics. In contrast, high-cost purified pharmaceutical grade CMC products are not necessary for applications in the construction, plastic, or oil industries instead of the cheaper ones (i.e., industrial-grade products). Additionally, mechanical strength, viscosity, and rheological properties also significantly restrict the application fields of synthesized CMC products. Therefore, the characterization of a CMC product is also an essential step for determining its further applications and marketization in different areas. Among the various characterization techniques, scanning electron microscopic (SEM) analysis [100], Fourier transformation infrared ray (FTIR) spectroscopy [101], and thermo-gravimetric analysis (TGA) [102] have been used. Although numerous research works have been published so far that introduce different uses and applications of CMC in various fields, all the findings are scattered throughout the literature. In 1945, Hollabaugh et al. [103] tried to summarize some of the application fields of CMC. Later on, in 2005, Heinze and his co-workers [104] exhumed an overview of carboxymethylated starch and cellulose production and their characterization techniques in their study. In 2017, Kukrety et al. [105] assembled the works of CMC synthesis from various waste biomass resources in their review. However, non-conventional waste resources of CMC, like waste paper, paper sludge, waste textiles, knitted rags, waste cotton linters from textile industries, and various conventional plant-based resources, were excluded from that study. Overall, there has been no major up-to-date review work where all the potential sources of CMC, their essential characterization techniques, and their uses and applications in numerous fields have been assembled and discussed together.

To envision this, herein, we discuss and assemble the reported works on CMC based on their sources, characterization, and applications in numerous fields like biomedical, pharmaceutical, construction, food and textile industries, energy production, water treatment processes, etc. Furthermore, synthesis roots of CMC from its various conventional (i.e., plant-based) and non-conventional (e.g., waste materials) precursors and their essential characterization techniques such as scanning electron microscopic technique, Fourier transform infrared spectroscopy, thermo-gravimetric analysis, etc. will be discussed here extensively. Furthermore, analysis of various physicochemical and mechanical properties of CMC products, like their rheological properties, viscosity, determination of the DS, DP, etc., will be elaborately discussed in separate sections. Thus, this work will provide an up-to-date and systematic overview of CMC from which researchers, scientists, or industry can quickly obtain the general up-to-date applications of this material at a glance, based on their sources as well as various critical properties, for example, viscosity, DS, and so on. Additionally, recommendations for further research works are also provided.

## 2. Characteristics of CMC

The answer of how CMC responds when it is used for a variety of conditions or applications is the central pivotal point to define the character of CMC. This section highlights the properties or parameters that directly impact CMC applications or behavior of the final products of CMC, such as rheology, viscosity, and DS. In contrast, rheology defines the physical properties and the flow and fracture behavior of the CMC’s final products (under different pressures). However, the rheological properties (stress-strain flow behavior, pseudoplasticity, viscoelasticity, and thixotropy) are primarily controlled by the viscosity. Likewise, viscosity is interrelated to the DS of CMC. Thus, the overall characteristics of CMC for various application purposes can be defined by the suggested three significant parameters (rheology, viscosity, and DS).

### 2.1. Rheological Properties

In between the discussion of the characterization of matter, rheology plays a vital role in connecting the study of the flowing behavior of matter and its deformation under the force of application. Moreover, the rheological study of materials gives an overall idea about the flow system like thixotropy, pseudoplastic, viscoelastic, and stress-strain flow behavior. After all, this behavior or properties of rheology are closely interrelated to the structure of polymer systems such as structure, particle size, concentration, shape or surface characterization, etc. According to the study of structure, CMC shows some complex and interesting flow behaviors under stress-strain action that directly impact the various application purposes of CMC such as food packaging, film fabrication, or coating of materials, etc. [106,107]. CMC’s thixotropy, pseudoplastic, or viscoelastic behavior is directly attached to suspension injection, paint, adhesive, food processing, cosmetics, etc. Here, the rheological characterization of CMC is discussed under the following subtopics.

#### 2.1.1. Stress-Strain Flow Behavior

For the application of CMC for various purposes, how or how fast CMC-based materials deform under applied force or various conditions are general questions that must be answered. Deformation study of materials is defined as the amount of strain under applied stress. The stress-strain relationship of materials to determine its flow behavior also helps to prescribe the CMC, whether it is suitable for a specified condition or not. As a polymeric derivative, CMC often behaves like a non-Newtonian fluid. CMC sometimes follows the property of Newtonian fluid or viscous flow behavior in low concentrations. Therefore, according to the Ghannam and Esmail (1997) investigation [34], CMC covers the Newtonian character at 1% and non-Newtonian above 1% (or 2–5%). The investigation followsthe shear stress-shear rate curves, which mainly were linear for all concentrations (1–5%), as shown in the Supplementary Materials (Figure S1a). Still, the viscosity-shear rate curves were nearly horizontal type (below or at 1%) or decreasing linear type (above 1% or up to 5%), as shown in the Supplementary Materials (Figure S1b). The horizontal curve indicates the flow is viscous or has a Newtonian character. Gradually decreasing curves define the shear thinning behavior (STB) or non-Newtonian character, indicating sharp and highest flow behavior. Other CMC properties like thixotropy, pseudoplastic, and viscoelastic behavior are fully interconnected to STB. Above all, STB plays an important role during the various applications of CMC, which control the film fabrication, packaging, injection molding, or melt strength property of CMC [34,107].

In the last decade, Benchabane et al. (2008) [108] noticed a critical shear rate (CSR) curve (ᵞ_c1_) in the viscosity-shear rate graph against all concentrations (0.2 to 7%) of the CMC polymer solution. According to the author’s discussion, the CSR limiting value helps define the change of the flow behavior of CMC. For example, below the critical concentration (<1% or 0.2 to 0.8%), CMC exhibits shear-thinning behavior; and above at 1%, CMC exhibits transition behavior (shear-thickening to shear-thinning) due to the gradually increasing shear rate. Moreover, in between ᵞ_c1_ < ᵞ < 1000 s^−1^, CMC exhibits Newtonian and non-Newtonian shear thinning behavior.

CMC’s flow study behavior, shear stress-strain behavior, transient shear stress (TSS) response, rheopexy (time-dependent shear stress), and yield stress of polymer solution are other necessary tests or parameters. The TSS response means a change in shear stress over a lower shear rate. Among these parameters, above 3% (i.e., 4–5%), CMC solution and rheopexy exhibit under the low shear rate (less than 10 s^−1^) and not otherwise [34]. Characteristics of rheopexy discuss the complex flows related to viscoelastic (linear and non-linear) behavior [109] and the thixotropic structure of CMC liquid [34,109]. Furthermore, the yield stress test of CMC provides information about the deforming ability of a CMC solution under applied stress. For example, CMC deforms easily, starts to flow, and maintains fluid behavior when the applied stress is crossed with the yield stress [34,109].

#### 2.1.2. Pseudoplastic and Viscoelastic Behavior

The pseudoplastic or viscoelastic properties of materials describe the flow behavior or response of the material under stress in an application purpose. Pseudoplasticity is defined as the shear-thinning behavior of fluid material. The CMC long-chain’s easy orientation under shear stress is the main reason behind the display of CMC pseudoplasticity. The viscosity gradually falls under the stress of the CMC and increases their flow behavior index. Moreover, the pseudoplastic behavior of CMC is affected by factors like DS value, molecular weight, and temperature. For example, pseudoplasticity is higher in high molecular weight and the DS value between 0.9 to 1.2 [34,109,110]. After all, CMC is widely applied in food processing (condensed milk, mayonnaise, etc.) or some cosmetics (lotion, toothpaste, etc.) for their pseudoplastic behavior.

On the other hand, the viscoelasticity of the polymer defines the possibility of exposure of both the viscous and elastic behavior of the polymer [107]. Furthermore, it is also defined as a time-dependent response (or relaxation time) under applied constant stress. The molecular structure of CMC is responsible for displaying viscoelasticity because CMC has a long chain, entanglement property, and high relaxation time. The response of viscoelasticity CMC polymers can be investigated by creep-recovery and dynamic test during application purposes. Among them, creep compliance, and elastic recovery index is calculated during the creep-recovery test to understand the response of viscoelastic behavior. Higher creep compliance, and flexible recovery index indicate the possibility of showing better viscoelasticity of CMC [34,108] because CMC is easily deformed by giving stress at higher creep compliance.

Moreover, the response of viscoelasticity depends on the concentration of the CMC solution. According to the proposal of Ghannam and Esmail (1997) [34] and Benchabane et al. (2008) [108], the viscoelasticity of CMC was above 3% and 2.5% concentration of CMC solution, respectively. Furthermore, at a low concentration of CMC (below 2.5 or 3%), CMC behaves as a viscous flow. However, above 3%, CMC acts like an elastic solid. Based on their viscoelastic behavior, CMC has been widely applied in a hydrogel form in the biomedical, drug delivery, and agriculture fields [111,112] as a stabilizer or thickener in food products [113] and so on.

#### 2.1.3. Thixotropy

Thixotropy is the most important property of non-Newtonian fluids and follows the STB [114]. Its behavior is mostly in contrast to the rheopexy illustrated in the Supplementary Materials (Figure S2a) [115]. CMC’s long-chain polymer demonstrates thixotropy for forming three-dimensional structures by intermolecular attraction. Generally speaking, thixotropy is defined as the transformation behavior of a shear-thinning system, where a high consistency gel is transformed to a low consistency solution. After a long time of rest, the thixotropic solution is again transformed into a gel form. CMC formulates a time-dependent hysteresis loop during the thixotropy behavior by two curves, like an up- and down-curve shown in the Supplementary Materials (Figure S2b) [110]. The up-curve indicates CMC’s high consistency or viscosity or defines the increase of shear rate against stress up to the desired value. After assuming the desired shear rate, viscosity, or consistency of fluid decreased with shear rate, this indicates the hysteresis loop’s downward curve. The thixotropy of CMC sometimes depends on its DS value or concentration factor. In the twentieth century, Feddersen and Throp (1993) [110] investigated the sharp thixotropy of CMC with low DS value (0.4 to 0.7) and high concentration or viscosity. During high concentration, thixotropy is higher than a lower concentration of the fluid due to the formation of higher insoluble regions in the fluid. According to Ghannam and Esmail (1997) [34], the thixotropy of CMC is uncountable with a 1 to 3% concentration of CMC solution. Above 3% (i.e., 4% or 5%), the thixotropy of CMC accelerates with an increasing concentration of CMC, whereas at 5% CMC, the solution shows higher and sharper thixotropic behavior than the 4% concentration of CMC. After all, CMC is widely used in various industrial applications as a thixotropic fluid, such as in pharmaceuticals (liquid or disperse drug, sustained drug release, drug coating, parenteral suspension, etc.); paints; adhesives; or foods. For example, in dispersing a drug, the thixotropy of CMC enhances a drug’s stability by gel-sol-gel transforms.

Moreover, in parenteral suspension injection, thixotropic fluid (CMC) plays an essential role in making the injection feel comfortable and accessible through the skin. When applying the pressure on the syringe plunger during injection, suspending the suspension particles are easily broken down and decrease their viscosity. A low viscosity (or consistency) of the syringe solution allows suspended drug particles to pass easily inside the skin without any panic effect. After injecting the drug, suspended particles are again aggregated and enhance their consistency inside the skin. This behavior also helps with the sustained release of drugs in the human body.

### 2.2. Viscosity

Viscosity is a measure of a fluid’s resistance to flow and defines the internal friction of a moving fluid. CMC is a polymeric material and provides a viscose solution in an aqueous medium due to its high solubility in water. Therefore, the viscosity parameter plays a vital role in describing the fluid behaviors, properties (thickening, emulsifying, etc.), and property-based applications of aqueous Na–CMC (or H–CMC). Because the high or low viscosity of the applied medium is maintained with increasing or decreasing CMC concentration, respectively; on the other hand, the viscosity property of CMC also depends on or is influenced by the source parameters (like cellulose particle size, molecular weight, and DS) and synthesis conditions (concentration of NaOH, reaction temperature, and pH of solution) of CMC from cellulose, as depicted in the Supplementary Materials (Figure S3) [10,116,117,118,119].

However, viscosity is considered the most critical parameter in various application purposes. For example, CMC is used in the food industry while the low viscosity type CMC acts as a moisture binder, and high viscosity type CMC acts as a gelation agent [120]. The lower viscosity-based CMC is used with sodium bentonite as a viscosity modifier or dispersion media in a drilling mud system. CMC increases the dispersion viscosity of bentonite and reduces the fluid losses in a drilling system [121]. Therefore in biomedical, tissue engineering, pharmaceutical, textile dye processing, foods, and cosmetic applications, the viscosity of CMC provides the recommended rheological properties. In soft tissue filler engineering, the lower-viscosity CMC hydrogels offer a softer feel via high spreading accessibility. Higher-viscosity CMC hydrogels give a firmer feel via less spreading accessibility [122]. For drug delivery purposes, CMC/MCC (microcrystalline cellulose) is used as biocomposite film-forming materials for drug delivery vehicles (such as hard capsules). CMC acts as a filler and improves the film’s strength by providing high viscosity [123].

Similarly, CMC has also been used recently to maintain the role of intrinsic viscosity of CMC/rosin/PEG (polyethylene glycol) drug nano-carriers [124]. CMCs are widely used in foods based on their viscosity characteristics and provide the recommended rheological properties, good texture, and mouth feel profiles. CMC is used as highly viscous materials in emulsified food or thickening juice [125,126]. Moreover, it is used in semisolid dairy products depending on their viscosity (low or high) to control the viscoelastic properties [66]. Due to control of the thixotropy and pseudoplastic behavior of cosmetics, CMC has been used with hyaluronic acid to develop a gel for application in cosmetics as CMC creates a better viscosity and texture profile in the gel [127].

### 2.3. Degree of Substitution

In cellulose chemistry, the reactive groups of cellulose are the most critical parameters in identifying their chemical activity. Three hydroxyls (–OH) reactive groups in anhydroglucose units of cellulose were introduced with the DS. The DS range is generally expressed for each unit zero to three. The DS value is technically defined as the average number of substitutes of the reactive group by other active molecules in the polymer chain. During the synthesis of CMC from cellulose, the value of DS is determined by the number of substituent groups (carboxymethyl) attached to each anhydroglucose unit.

The DS plays a vital role in the case of CMC properties as the solubility, emulsibility, thickening property, acid resistance, viscosity, stability, and salt tolerance properties of CMC depend significantly on the DS value [128]. For example, the CMC polymer is entirely insoluble but is swellable under the 0.4 DS value. In contrast, CMC is fully soluble beyond 0.4 (DS) [11,119]. The increasing solubility with DS has increased carboxymethyl substitution and substitution uniformity along the macromolecular chain [129,130]. In another article, D.N. S. Hon (2001) [131] proposed that CMC with DS values from 0.1 to 0.4 and 0.5 is soluble in cold and normal alkali (4–8% NaOH) solution, respectively. Over and above, the decreasing particle size and increasing DS value of CMC affects the viscosity-increase [119], salt or alcohol tolerance, and enhancing hygroscopicity [132]. Therefore, CMC’s crystallinity is also related to DS, while the crystallinity was demonstrated under the DS of 0.82 and disappeared beyond 1.0 [129]. The degree of CMC substitution also directs the properties of cellulose substrates. Controlling DS allows the balance of properties between CMC and cellulose substrates [30,31,32].

However, many researchers have reported on the synthesis of CMC in the last decade. It is noteworthy that depending on the source of CMC’s cellulose and synthesis procedure, the DS value of CMC varies from one to another. For example, based on different sources, the recommended DS values at optimum condition are 0.17 (*Musa paradisiacal* fruit) [133]; 0.28 (*Musa parasidiaca* stem) [133]; 0.29 (oil palm fiber) [134]; 0.31 (palm kernel cake) [134]; 0.33 (*Tithonia diversifolia* stalk) [133]; 0.35 (*M. sinensis*) [135]; 0.3–0.4 (cotton fiber) [30,31,32]; 0.51 (seaweed) [136]; 0.67 (sugar beet pulp) [137]; 0.76 (*C. papyrus*) [135]; 0.80 (*E. crassipes*) [135]; 0.82 (sago waste) [5]; 0.87 (durian rind) [9]; 1.07 (office waste paper) [19]; 2.39 (corn leaves) [138]; 1.21 (waste disposable paper cups) [139]; 1.76 (water hyacinth) [140]; 2.41 (corn husk) [119], etc. On the other hand, synthesis techniques including the range of concentration of reagents (NaOH, MCA) or reaction temperature and time, significantly impact the DS values. In particular, the value of DS can be fine-tuned by the concentration of reagents like isobutanol, ethanol, NaOH, and etherification agent (MCA). For example, depending on the varying concentration of NaOH, V. Pushpamalar (2006) [5]; P. Rachtanapun et al. (2012) [9]; A. H. Saputra et al. (2014) [140]; M. S. Yeasmin et al. (2015) [119]; and Ibikunle et al. (2020) [141] reported a range of DS of 0.51–0.82 (for sago waste) [5]; 0.56–0.87 (for durian rind) [9]; 0.14–1.76 (for water hyacinth) [140]; 0.1–2.41 (for corn husk) [119]; and 0.15–0.93 (for African star apple seed shell) [141], respectively. A higher DS value was determined mostly against 30% NaOH [116,119,141] and negligible at 25% and 10% NaOH [5,140].

Interestingly, under or above the 30% NaOH, the DS value often declines due to low reaction rate and polymer degradation [116]. Thus, 30% NaOH has been considered to be optimum for CMC synthesis from various sources with good DS values. Similarly, several research articles have demonstrated that during the etherification of cellulose, changing the concentration of MCA and various solvents (isobutanol, ethanol, etc.) has impacts on DS values [5,19,119,138,140]. For the carboxymethylation of cotton cellulose, the increase in the concentration of monochloroacetic acid-enhanced DS [30]. The effect of reaction time and temperature on DS has been reported by G. Joshi et al. (2014) [19], while DS increased up to 50 °C (optimum temperature) and 3 h (optimum time) reaction time. The DS decreased beyond the optimum temperature and time due to improving oxidant atmosphere and oxidative degradation of CMC. This similar reaction time (3 h) has been validated by V. Pushpamalar (2006) [5] for an optimum DS value against 45 °C. However, in 2015, M. S. Yeasmin et al. [119] used a prolonged reaction time (3.5 h) and a slightly higher temperature (55 °C) in comparison with other researchers and optimized their process to achieve a higher DS value from corn husk cellulose.

Furthermore, the effect of starting material (cellulose) particle size on DS has been reported by MS Yasmin et al. (2015) [119] and MS Rahman et al. (2020) [142]. According to their investigations, the highest DS was found with the smallest particles of cellulose; for example, a DS of 2.41 was found with 74 µm while the decreased DS of 1.83 was observed with an increasing particle size cellulose of 100 µm. As reported by the authors, a smaller particle size of cellulose imparts a higher surface area than larger particle size, which facilitates the high degree of collision between the reactant and cellulose particles; thus, the more hydroxyl groups are easily substituted by the carboxyl group. According to the author’s claim, they obtained the highest DS value in a single step instead of many actions, making the low process cost, less time-consuming, and more eco-friendly than those reported in other papers [119,142].

CMC has wide applications in different fields depending on their DS values. For example, C. Arthur (1989) [143] synthesized CMC with a DS value of 0.5–1.2. Between this range, CMC is widely used in food additives, paper size, paints, coatings, detergents, and oil-well drilling muds. In contrast, Coffey et al. (2006) [144] and Baiqiao et al. (2009) [145] published an article on the synthesis of CMC with a DS value of 0.6–0.95, which has the greatest use in the food industry. However, in 2011, based on the “Joint FAO/WHO Expert Committee on Food Additives, 2011” principle, Casaburi et al. (2018) [128] also proposed CMCs with a DS interval of 0.2–1.5 for food applications. Among the various uses of CMC in food applications, CMC is widely used to increase the durability of acidic milk drinks. CMC has been used as a stabilizer and improves the stability of milk; this property is ameliorated with increasing DS [145] because increasing DS values improve the electrostatic repulsion in casein particles, reducing the milk sedimentation or phase splitting [68]. Moreover, Parikh et al. [31,32] developed cotton burn dressings by partially exchanging sodium cations with silver cations from sodium carboxymethyl cotton for wound dressing or drug delivery purposes. The resulting carboxymethyl gauze/nonwovens with DS of 0.3–0.4 retained a greater amount of silver nitrate solution for better antimicrobial treatment. CMC is used as a hydrogel form with a proper DS value. For example, P. Komorowska et al. (2017) [146] synthesized CMCs with DS values of 0.62–0.79 to make a strong synergism-based Na-CMC/propylene glycol/H_2_O hydrogel.

Furthermore, in developing the protein–CMC complex, the DS of CMC is an essential factor. During complexation, on the one hand, higher DS inhibits the complex formation. On the other hand, a lower DS provides a better complexation environment between protein and CMC. Y. Wang et al. (2019) [147] reported the highest optical density (higher LPI-CMC complex form) with low DS (0.7) during the interaction between lentil protein isolate (LPI) and CMC.

On the other hand, depending on the suitable DS value, CMC is used in lithium-ion batteries as a suitable binder in the anode. BR Lee and ES Oh (2013) [148] demonstrated CMC as a binder for a Li_4_Ti_5_O_12_ (LTO) anode with an optimum DS vale (1.2). High DS (1.2) improves the strong binding capacity between LTOs, has high ion conductivity, good lithium-ion mobility in the cell, and provides the best cell performance. After all, the DS value of CMC provides an overall performance of the property of CMC. This section has critically reviewed the importance of DS and how the values of DS vary depending on the starting material, reaction conditions, physical factors, for example, particle size, etc.

## 3. Synthesis of CMC from Its Various Sources

### 3.1. CMCs from Various Plant-Based Precursor Materials

From the earliest production of CMC, the terrestrial precursors of cellulose have been used most of the time. However, the concomitance of other compositional essences such as lignin, pectin, hemicellulose, and minerals without the expected cellulose extent demands excess energy input and costs for their removal with some excessive pre-treatment steps. Consequently, the use of conventional terrestrial cellulosic precursors is losing interest day by day in CMC production due to their limited availability and expensive cost-expanding pre-treatment steps. Many researchers have reported a moderate amount of cellulose percentage (i.e., 31~60%) in numerous agricultural by-products and wastes such as fruit peels, straws, corn cobs, leaves, etc. [149]. Therefore, an emerging interest has grown in recent years to utilize these materials in commercial CMC production for various applications, according to the properties of the obtained CMC. It is worth mentioning that the synthesized CMCs from different plant-based or agricultural precursors may not show similar physiochemical or morphological characteristics (e.g., DS, rheological properties, viscosity, water, and oil retaining capacity, etc.). Therefore, their targeted fields of application may vary from each other. To improve their existing properties or add new properties, the production of CMC from cotton fibers, which consist of about 95% cellulose, has been carried out [30,31,32].

A significant advantage of plant-based precursors is their high availability in different regions around the world. Compared to the commercial precursors of CMC (such as wood), they are more highly available in other areas of the world at a negligible cost, or sometimes even free of charge. For instance, Meenakshi et al. (2002) [150] reported banana pseudo-stem (i.e., an agricultural waste) as a potential source of cellulose. A few years later, Adinugraha et al. (2005) [10] synthesized a technical grade CMC (of 98.23% purity) from this agricultural waste. The following year, Pushpamalar et al. [5] demonstrated a facile synthesis of commercial-grade CMC products from sago thwacks, which are the waste by-products of various food industries. Bamboo shavings are waste by-products of bamboo industries that contain a considerable amount of cellulose (i.e., 33–45%), along with lignin, protein, hemicellulose, and pectin, as well as some other minor extracts [151,152,153].

Nevertheless, many of these materials are often incinerated or discarded from the industries every year. A few years back, Chen et al. (2014) [117] synthesized technical grade CMCs from abundant waste materials that possessed diversified viscous properties as well as DS values. Flexibility in such properties made it a highly promising precursor for the industrial production of CMC for application purposes. Corncob is considered a waste material after removing the seeds of maize (*Zea mays*), which is abundantly grown in different regions worldwide. According to Sing et al. (2012) [6], around 16,780,000 tons of maize cereals were produced annually only in India during 2011. Therefore, the authors paid attention to this plentiful waste for synthesizing CMC and obtained a high-quality product with a moderate DS value (i.e., 1.18). A fibrous, low-protein by-product of palm oil cultivation industries (i.e., oil palm fronds) has been proposed as another low-cost precursor of CMC. In 2015, a rough annual production of about 164 million tons of oil palm fronds was reported in [154]. In that study, the author synthesized a high purity CMC product with a high production yield (i.e., 170.1%) and a moderate DS value (i.e., 1.1) from this low-cost precursor through the conventional alkylation-etherification process. Water hyacinth, a free-floating abundant invasive plant of aquatic environments, can be utilized as an effectual precursor of CMCs [140]. A noxious weed (i.e., *Lantana camara* L.) that can impart various negative impacts on land productivity and consequently, even on the biodiversity of the total ecosystem, was also reported as an influential precursor for the synthesis of a non-Newtonian pseudoplastic CMC product with high viscosity and moderate DS value (i.e., 1.22) by a research team [11]. Similarly, the feasibility of CMC synthesis from numerous plant-based precursors has been demonstrated in dozens of studies by multiple researchers. A list of such adequate studies has been summarized in the Supplementary Materials (Table S1).

### 3.2. Production of CMCs from Non-Conventional Precursor Materials

Aside from conventional plant-based cellulosic precursors, some waste materials of the textiles industries (e.g., knitted rag, cotton linters) as well as regularly used household and office products (e.g., office waste papers, paper sludge, waste textiles, etc. are available almost free of charge, which can be utilized for the synthesis of CMCs. Production of CMC and its various composites for numerous applications from these waste materials reduces the production cost and plays a vital role in preventing environmental pollution. For instance, cotton linters are a waste product of cotton cleaning factories. In 1954, Ott et al. [155] found that almost 3–5% of cotton fibers are wasted as cotton linters, which contain a high percentage of cellulose (i.e., about 90%) in their raw states. Hence, they can synthesize different grades of CMC products for numerous applications. A few years back, Jahan et al. (2007) [23] reported a facile synthesis of water-soluble CMC from cotton linters that demonstrated an appreciable DS value with high viscosity. In another study, Hivechi et al. (2015) [156] reported a cost-effective, environmentally friendly process for the synthesis of CMC from cotton linters using ultrasonic and microwave radiation that facilitated the conventional approach (i.e., alkylation-etherification) of CMC production. Fakrul Alam and Mondal (2012) [20] synthesized carboxymethylated cellulose from the knitted rag, which is a common waste material of almost all textile industries and contains a high percentage of ɑ-cellulose (e.g., 95–98%). To investigate the effect of multiple carboxymethylation steps on the CMC quality and grade, the authors conducted consecutive seven-step carboxymethylation using the same experimental conditions and chemicals listed in the Supplementary Materials (Table S2). Multiple carboxymethylation steps of the crude cellulose demonstrated high yield (%) of CMC, DS, molecular weight, and water solubility. More specifically, in the first carboxymethylation, the DS value and yield of CMC obtained were only 0.91 and 360%, respectively. In contrast, after repeating the process seven times, these values reached up to 2.84 and 1494%, respectively.

Recently, Li et al. (2020) [157] synthesized CMC from office waste papers to fabricate an environmentally friendly, low-cost, crust-dust suppressant. However, a few years earlier, Joshi and his co-workers (2015) [19] demonstrated the feasibility of CMC synthesis from mixed office waste papers after deinking and pulping them. It is worth mentioning that deinking is an ancient process for reusing waste papers. The ink particles are removed from fiber surfaces either by chemical treatment methods such as alkali boiling [157]. The dispersed ink particles are removed from the fiber suspensions by the floatation method [158]. Therefore, a thousand tons of waste paper daily obtained from various offices and industries can be utilized as a plentiful precursor of CMC. Table S2 in the Supplementary Materials assembles some of the most promising research works based on the synthesis of CMCs from such waste cellulosic materials. We have summarized their grave essences in brief.

### 3.3. Synthesis Route of CMC

The conventional method for CMC synthesis is the alkylation-etherification process. In every research work related to the synthesis of CMC, this idiosyncratic process has long been used from the very beginning of CMC synthesis from various cellulosic precursors. The only difference between these works is in the applied ratios of the affined chemicals to the cellulosic extents of the precursors and variation in the reaction parameters such as temperature, reaction time, pH, etc. It is noteworthy that all of the natural and industrial cellulosic precursors (e.g., leaves, stems, pulps, paper wastes, microbial as well as various textile-based precursors such as cotton linters, knitted rags, etc.) may not possess similar compositional characteristics that can provide a fixed amount of cellulose extract every time. Most precursors contain a considerable amount of lignin, pectin, ash, and other minerals (e.g., phosphorous, potassium, calcium, etc.) besides the cellulosic percentiles. Therefore, on occasion, some pre-carboxymethylation steps such as proteolysis (treatment with protease enzyme), inactivation of various enzymes of the plant-based precursors (e.g., deactivation of pectic enzymes present in orange peels) [14], delignification, dewaxing [140], bleaching, defatting [14], and acid washing, as well as treatment with some essential chemical compounds (e.g., methanol, ethanol, chloroform, etc.) [11,13,159] and removal of hemicellulose [140] are carried out for the extraction of pure ɑ-cellulose from natural sources.

**Scheme 1:**
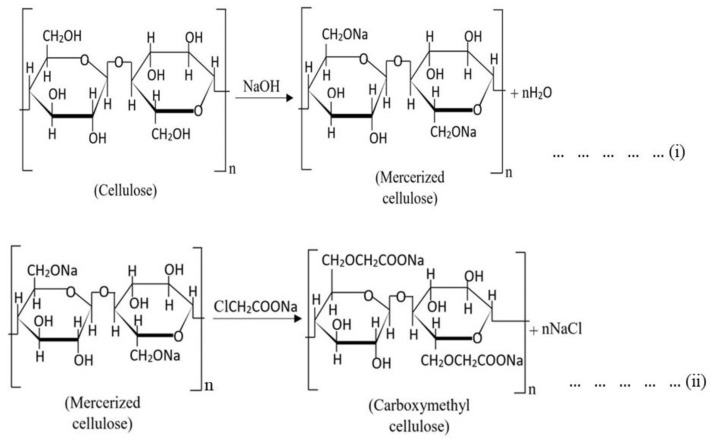
Chemical reactions of CMC synthesis from cellulose [6,7,19,31,32,157].

Carboxymethylation of the pure ɑ-cellulose is performed basically in two significant steps. In the first step, the pre-purified cellulose extracts are mixed with alkali reagents (e.g., NaOH) in a reaction vessel for a specific time. During this time, the pure cellulose contents are mercerized. Subsequently, –OH functional groups from each AGU unit are substituted by –ONa groups targeted for the posterior substitution by carboxymethyl groups in the etherification step (Scheme 1, reaction (i)). Concentrations of the alkali, ratios between the alkali and cellulose, and reaction temperature should be maintained very carefully. A slight change in these parameters can significantly change the final product’s DS value and other physicochemical properties. An inert solvent (such as ethanol [31,32,160], 2-propanol [161], isopropyl alcohol [6,22,162], or isobutyl alcohol [137], etc.) is added in this step as a diluent as well as a swelling agent that facilitates the penetration of the affined reagents into the cellulose structure.

(i) An etherifying agent is added into the reaction vessel in the next step (i.e., etherification). NaMCA is often used as an etherifying agent [7,10,19,157,163]. However, some researchers have modified their process using other reagents, such as diazomethane [15], instead of the conventional etherifying agent. The mercerized cellulose is stirred continuously with the reagent for a certain time. An optimized temperature is required to obtain the best result, and therefore, the reaction temperature should be maintained carefully. During this step, the reactions can be represented as reaction (ii) (Scheme 1).

**Figure 2:**
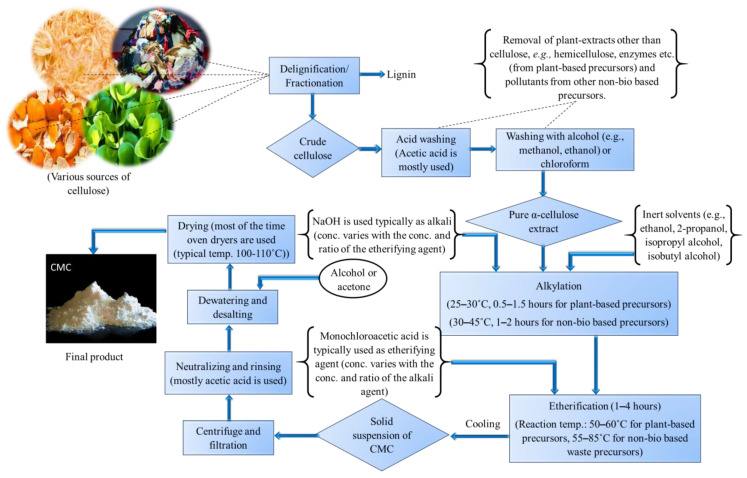
Schematic representation of CMC production from its various precursors.

After mercerization and etherification, a solid suspension of CMC is obtained. Then, after ensuring that the pure product is free from other impurities, the suspension is then centrifuged and filtered with a congruous filtration medium [5,8,14,15,18,19,163]. The product is then polished and finalized by performing some post-treatment procedures such as neutralizing with weak acids (e.g., primarily acetic acid is used [9,14,157,160]), washing with alcohol for dewatering and desalting [7,10,16,20,139] and/or acetone [6,163], and finally drying the obtained solid product at a specific temperature (mostly, oven drying is carried out rather than sun drying to maintain the temperature correctly. A flow diagram depicting the entire process of CMC production from its various precursor materials is shown in Figure 2.

#### 3.3.1. Factors Affecting the Characteristics of CMC

##### The Temperature of the Etherification Process

The temperature of the etherification process plays an important role in the DS, degree of polymerization (DP), and other physicochemical properties such as viscosity, swelling behavior, thixotropy, etc., of the final product. The product’s best DS value is obtained only at a specific temperature. For instance, Pushpamalakar et al. (2006) [5] obtained the highest DS of their products (0.821) at 45 °C reaction temperature. Above and below this temperature, the DS value decreased significantly. Golbaghi et al. (2017) [16] obtained the best DS value of their sugarcane bagasse-derived CMC product at 57.85 °C after conducting dozens of reactions at different temperatures from 30 °C to 70 °C.

Similarly, Singh et al. (2012) [6] reported the highest DS value of their corn cob-derived CMC products only at 60 °C temperature. Silva et al. (2004) [164] investigated the effect of reaction temperature on the carboxymethylation of cashew tree gum-derived cellulose. DS value of their carboxymethylated product diminished considerably (i.e., from 0.75 to 0.16) as the reaction temperature was enhanced up to 70 °C from 30 °C. However, the reaction yield was increased (i.e., from 32% to 57%) at the same environmental conditions. Hence, the optimum temperature is not the same for every terrestrial or other cellulosic precursors. However, they introduced their reaction optimizing tendency within a specific range of temperatures. Outcomes of various studies revealed that most carboxymethylation reaction temperatures were optimized at 50 °C–60 °C for terrestrial precursors of cellulose [6,7,9,10,11,13,16,161]. However, some exceptions have also been reported in other studies [5,14].

On the other hand, the optimum temperature crossed over 70 °C for the carboxymethylation while using various non-biological waste materials as a precursor of CMC [19,20,139,157]. This phenomenon can be explained by facilitating the forward reaction kinetics of carboxymethylation as the temperature was raised to the optimizing point. Once the temperature is presented to an end, the posterior aggrandizement of the weather increases the reaction kinetics. Still, it induces the degradation of the cellulose structure in the presence of atmospheric oxygen, which diminishes the CMC production automatically [5,6,19]. Therefore, the optimum temperature for carboxymethylation largely depends upon the precursor of cellulose to obtain the best production yield, and it is crucial to maintain the reaction temperature properly.

##### Dosage of the Etherifying Agent

The dosage of the etherifying agent has a significant impact on the final product’s physicochemical properties and DS values. Casaburi et al. (2017) [128] synthesized a highly purified CMC from bacterial cellulose where the *Gluconacetobacter xylinus* strain was used as the precursor. The authors demonstrated a significant enhancement in the DS value while gradually increasing the dosage of an etherifying agent (NaMCA) per mole of AGU. However, after a certain point (i.e., NaMCA: AGU = 2:1), the DS value started to decline. Similar results have also been found by some other researchers. A few years ago, Joshi et al. (2015) [19] received the highest DS value of their product (1.07) by using 0.11 M NaMCA as an etherifying agent. Afterward, using both the higher or lower concentrations of the reagent around this value significantly reduced the DS of the CMC. Almost similar results were reported by Pushpamalakar et al. (2006) [5] while synthesizing CMC from sago wastes (i.e., a by-product of the sago starch production industries). They obtained the maximum DS value (0.77) of the synthesized CMC using 6 g of NaMCA per gram of the precursor as an etherifying agent. Upgrading or reducing the dosage of NaMCA from this point diminished the DS value. Such a phenomenon can be attributed to the fact that enhancing the dosage of the etherifying agent up to the optimum point enhances the availability of the acid molecules in the proximity of the cellulose hydroxyls, thereby facilitating the carboxymethylation [165]. Later, the addition of an excess amount of NaMCA over this optimum value brings no payments at all due to the exiguity of the mercerized cellulose alkoxides that can react with this etherifying agent [11]. However, the redundant chemicals then react with the available mercerizing agent (i.e., NaOH) in the reaction vessel, form a by-product (sodium glycolate) presented in reaction (iii), and consequently reduce the rate of the significant carboxymethylation reactions [11]:

NaOH + Cl-CH_2_COONa → OH-CH_2_COONa + NaCl … … … … …(iii)

##### The Concentration of the Alkali

The concentration of the alkali used for the mercerization of the cellulose (i.e., NaOH) also plays a vital role in ascertaining the DS of the final product. A few years back, Mastrantonio et al. (2014) [163] synthesized a technical grade CMC using short cellulose fibers from paper industry effluents. They experienced a considerable reduction in both the DS value and yield of the final product when the concentration of NaOH was enhanced from 7.0 g/mL to 10.5 g/mL. Similar outcomes have also been demonstrated by some other researchers. For instance, Bhandari et al. (2012) [166] reported that the DS value of their carboxymethylated product decreased from 0.56 to 0.46 when the alkali dosages were increased by 20 g.

Furthermore, a noticeable reduction in reaction efficiency was also experienced. More specifically, the reaction efficiency decreased from 20.12% to 16.39% as the dosage of NaOH was increased from 30 g to 50 g, respectively. Pushpamalakar et al. (2006) [5] reported that increasing the concentration of NaOH up to a certain amount (i.e., 25%) enhanced the DS value of their sago waste-derived CMC. However, the posterior enhancements in this concentration caused a considerable reduction in the DS. Casaburi et al. (2017) [128] obtained an increasing DS of their bacterial (*Gluconacetobacter xylinus*) cellulose-derived CMC while it enhanced the alkali concentration up to an optimizing point at the mercerization step. Similar results were also reported by Rachtanapun et al. (2011) [159] for their *Mimosa pigra* derived CMC films and powders. Although their product’s DS value was enhanced considerably up to a 50% NaOH concentration, it suddenly started to drop from higher concentrations of alkali.

However, the key factor behind these phenomena is the alkali:etherifying agent ratio applied in the reaction procedure. It is only at a certain ratio between the alkali (NaOH) and the etherifying agent that the highest DS value and the best physicochemical and mechanical properties (e.g., tensile strength, water retention capacity, viscosity, elongation, etc.) are obtained. This is said to be the optimized ratio of a certain cellulosic precursor. Whenever the respective dosage of mercerizing or etherifying agents increases or decreases, a competitive side reaction (reaction (iii)) between these two reagents predominates the major alkylation and etherification reactions. Consequently, an unwanted by-product (i.e., sodium glycolate) is formed that leads to less polymer degradation as well as substitution of the new functional groups into cellulose structures (i.e., reduces the DS value) [22,151,164].

Moreover, the concentration of NaOH and the etherifying agents also affects the other physicochemical properties of the carboxymethylated product, such as viscosity, water retention capacity, etc. Rachtanapun et al. (2011) [159] synthesized CMC films from an invasive weed (i.e., *Mimosa pigra*). Each of the films synthesized using different concentrations of NaOH in their mercerization step exhibited significant differences in water vapor transmission rate and water vapor permeability in experimental conditions (e.g., 25 °C, two days, fabricated film area 28.27 cm^2^). Both properties increased with the increase in NaOH concentration. Varshney et al. (2006) [11] showed that the viscosity of their carboxymethylated product derived from *Lantana camara* (a noxious weed) was increased, as apparently, the concentrations of the mercerizing agent (i.e., NaOH) increased up to a certain point. However, the posterior enhancement dropped off with the alkali concentrations. Similar results were also reported by Rachtanapun et al. (2012) [9] and Adinugraha et al. (2005) [10].

This phenomenon can be explained as the introduction of carboxymethyl functional groups into the cellulose structure increases the hydrodynamic volume of the product, which results in a gradual enhancement of its viscosity. The DS (i.e., the introduction of carboxymethyl groups into cellulose) is highly dependent on NaOH concentration [5,128,159,166]. Thereby increasing the concentration of NaOH ultimately increases the viscosity of the product. However, after a certain point, when the hydrodynamic volume of CMC surpasses its maximum limit, applying more dosage of NaOH or increasing its concentration results in the degradation of the cellulose structure [11]. Therefore, optimizing the alkali and the etherifying agent’s concentrations and ratio should be the priority in obtaining the best outcomes from the conventional alkylation-etherification process.

## 4. Application of CMC

### 4.1. Application in Textile Industries

Over the years, various polysaccharide-based thickeners have been widely used in textile printing with several types of dyes and fabrics such as guar gum, tamarind, corn starch, tapioca starch, etc. CMC has recently been developed as a very effective thickening agent in paints and textile varnishes for its better water-absorbing property. For example, the synthesized CMC from lignocellulosic waste was used as a thickener in vat dyes for textile printing [57]. Modulating the thickening property of vat dyes controls the proper viscosity for yielding a good quality paint. It can also hold the dye particles in the printing area with other chemicals or printing assistants. Furthermore, it acts as a good vehicle for dye materials and promotes better binding into the textile specimens. Last year, Fangfang An et al. (2020) [167] reported the rheological properties and performance of CMC or CMHPC (carboxymethyl hydroxypropyl cellulose-CMC derivative) as a thickener more densely in the case of the printing of high-quality reactive dyes.

Based on the high-viscosity and film-forming properties, CMC has been proposed to harness many purposes in the textile industry. Due to having a water solubility property, it has been highly suggested for use in printing pastes, sizes, finishes, and lubricants. Furthermore, in textile fabrics, crude CMC occupies a vast position and acts as a sizing agent, including the sizing of filament threads, yarns, and other textile materials. The low toxicity, biocompatibility, water solubility, and ease of removal by the washing property enhance its priority in textile sizing applications. For example, CMC and starch-based hybrids act as sizing agents in cotton fabrics or yarns [59]. CMC acts as an additive, increasing the adhesion of starch sizes to cotton and cotton blends. Moreover, CMC often applies to textiles as a sizing agent in the form of CMC-g-polyvinyl alcohol, CMC-g-polymethyl methacrylate, etc. [20,60].

Moreover, in silk fabrics, CMC is applied as a pre-treatment and leveling agent to improve the color fastness and adjust the color difference in digital printing. During textile printing, CMC controls the viscosity of color with a mixture of other agents and enhances the dye’s hydrophilicity and penetration [88], which provide better sharpness and printability of color. For example, the CMC/sodium alginate/dextrin mixture proposed by Dong-Seok et al. (2013) [58] was used for the digital printing of textiles. On the other hand, Kolmana et al. (2017) [168] developed CMC-based silica/polyelectrolyte complexes for painting applications. During canvas painting on textiles, CMC enhances adhesion between the complex and cotton fiber, which improves the strength of the materials. In cotton fabrics, the introduction of CMC increased water (or antimicrobial liquid) retention for absorbent and wound dressings [30,31,32]. Such newly added properties can be achieved without losing fiber integrity and carding ability using an appropriate CMC substitution. As a result, carboxymethylated cotton fibers are applicable in the fabrication of various personal hygiene and wound care nonwoven products.

Furthermore, CMC hydrogels have taken a broad position in textile waste management, where CMC hydrogels are used as an environmentally friendly adsorbent for removing dyes from wastewater or solution. Various researchers have proposed different CMC-based hydrogels due to the variation in pigments (likes organic or azo dyes, anionic or cationic dyes, etc.). This is broadly discussed in the wastewater treatment part of this review article. Here, one example is disclosed for demonstration as part of the textile application. Like, recently, Kokkarachedu and his fellows (2017) [169] formulated a CMC/acrylamide/graphene oxide hydrogel, which is used for the removal of organic-based dye (Acid Blue-133) from waste liquor via the adsorption method.

A few CMC composites have been examined as bioresorbable textile materials for rapid and efficient hemostasis purposes in modern medical textiles. Recently, Suchý et al. [170] proposed a CMC/hyaluronic acid (HA) or CMC/HA/etamsylate composite-based (with or without active ingredient) hemostatic nonwoven textile for faster blood clotting and wound healing. To provide proper therapeutic care in the human body, the release of active ingredients from medical textiles was also controlled using CMC as the coating material. This information was applied by Roy et al. (2017) [171], and they developed a chitosan/CMC/glutaraldehyde-based microcapsule for application on cosmetic textiles. Furthermore, during wet wound care, the CMC (as an acid form) is used in combination with collagen (collagen/CMC) to form nonwoven textiles [172]. CMC provides excellent mechanical properties during application and ensures safer wound care in wet conditions. Moreover, to monitor health and biomedicine activity, CMC is used as carbonized CMC (smart fabrics) on flexible electronics [173].

However, CMC derivatives are also being introduced in textile products due to their antimicrobial activity. Likewise, Ebru Bozaci and his co-workers (2015) [61] fabricated cotton fabrics by a fumaric acid/CMC-based silver nanocomposite, a safe, hygienic, comfortable, biodegradable composite, and has excellent antimicrobial activity. In the fabrication of nanoparticles, CMC acts as a stabilizing and reducing agent and reduces the silver nanoparticles by hydrolyzing CMC. In the current year, CMC was used as a coating material on nonwoven fabrics and developed a better or favorable antimicrobial textile with the presence of AgNPs (silver nanoparticles). This type of antimicrobial-based nonwoven textile was tested on rat skin (for wound healing) by Montaser et al. (2021) [174].

Additionally, some CMC derivatives have been formulated by introducing different antioxidant or antiradical materials. Thus, they are widely used in wound healing application-based textile materials. Krizova and Wiener (2013) [62] formulated polyphenols (antioxidant or antiradical material) and CMC-based fresh gel for textile materials. Generally, this gel is applied to wound healing textile cotton for the protection of healing cells from oxidative damage, where CMC acts as a controlled release carrier of polyphenols and stabilizers in textiles.

In modern times, smart or intelligent textiles increased as priorities in the textile industry’s revolution. Innovative materials are defined as environmentally responsive textiles like thermosensitive, pH-sensitive textiles, etc. Additionally, CMC or its derivatives are used as part of the component in the production of innovative materials. According to the research by the Chinese and Japanese, CMC can be used in thermosensitive textiles in a hydrogel form like the photographed CMC/acrylic acid/poly-N-isopropyl acrylamide (PNIPAM) hydrogel [175], which is used in innovative fabrics for formulating thermosensitive water-absorbing features of materials. The presence of toxic PNIPAM has a poisonous effect in this thermosensitive hydrogel, which is very problematic. Due to the poisonous effect of PNIPAM, Selestina and Vanja (2011) [63] proposed a new non-toxic, biodegradable and, pH and thermosensitive CMC-based hydrogel to fabricate knitted cotton fabric. During fabrication, this hydrogel formed a thin layer on the textile fabric surface, which showed better absorbance sensitivity in the different pH solutions and changing temperature when the fabricated textile was drowned in solution, where the concentration of CMC controls the pH or thermosensitive property of the hydrogel. Recently, a three-layer-based (chitosan/CMC/indicator dye) non-invasive, biocompatible, comfortable, and sweat pH-lactate sensitive textile was examined by Promphet et al. (2019) [176].

**Figure 3:**
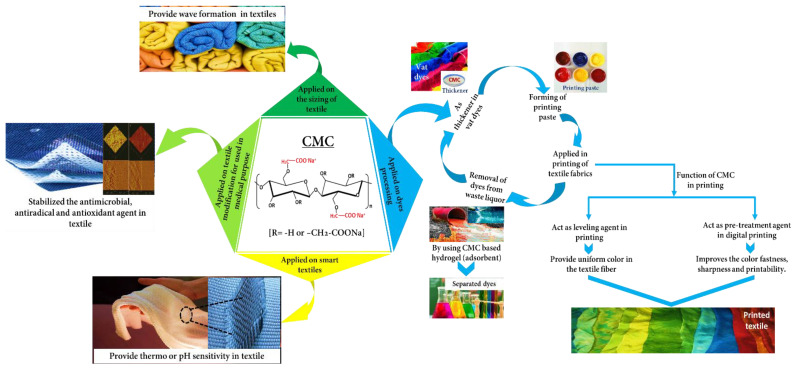
Schematic representation of the textile-based CMC application.

Various textile-based CMC applications have been shown by many researchers focusing on thickeners in dyes, pre-treatment or film-forming or sizing agents in fabrics, adsorbents for dye removal, its derivatives or composite form in antimicrobial or antioxidant purposes, and intelligent textiles. However, these properties were hardly combined in a single review paper showed in the Supplementary Materials (Table S3). Therefore, herein, we gathered up-to-date reported CMC data for textile applications and critically reviewed and summarized it, especially emphasizing four parts such as dye processing, sizing of the textile, modification of medical-based materials, and bright fabric (Figure 3). Additionally, we unveiled the specific function of CMC for use in textile applications that would be worthy for people interested in this field.

### 4.2. Application in Food Industries

**Figure 4:**
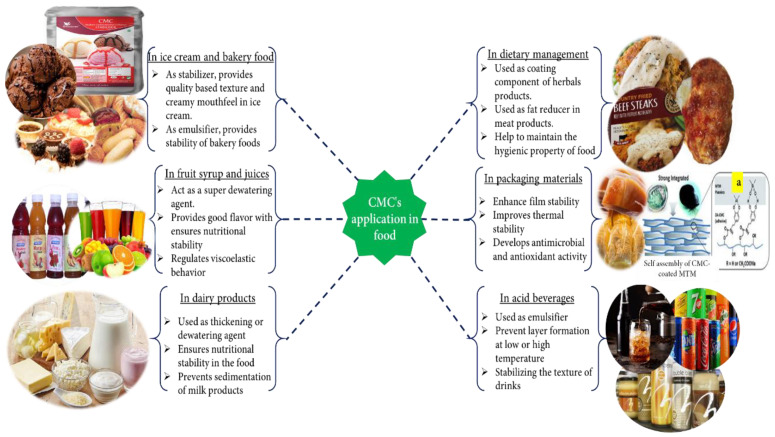
Various applications of CMC in food. (**a**) Assembly of CMC in coating composition [198]. Reproduced with permission from [198]. Copyright 2021, Royal Society of Chemistry.

The food industry plays an essential role in providing food to human society. There are many auxiliary agents, for example, various polysaccharide (alginates, gums, agar, some pectin and galactomannans, modified starches, modified cellulose, CMC, etc.) [177,178], hydrocolloids like soluble soybean polysaccharide (SSPS), sugar beet pectin (SBP), and xanthan gum powder [177,179] that are used in the food industry as a binder, thickener, fixing agent, and emulsifier to make quality foods. CMC is frequently used in the food industry (Figure 4) as an auxiliary agent due to some of its excellent properties, such as being odorless, tasteless, noncaloric, physiologically inert, forms a clear solution without opacity, preventing the capacity of gravitational separation of suspended particles, etc. [180,181].

These CMC features help improve food quality and the desired good mouthfeel to ensure food safety. Usually, CMC is used as various auxiliary agents in the food industry, such as thickeners, emulsion stabilizers, additive stabilizers, moisture binders, suspending and improving texture, water retaining (or dewatering), etc. Additionally, CMC is used to fine-tune the rheological property, structure, flavor, and appearance of products and their pseudoplastic properties. In addition, it is used as a coating or packaging material to ensure the long time safety of food products [182]. Many studies have appeared on the use of CMC in food products in the literature. For example, CMC is used as a thickener in nano-emulsions based on olive oil to improve the physical property and stability via controlling particle size, concentration, and texture [125]. Depending on the viscosity, CMC is used in the food industry. Namely, low viscosity type CMC acts as a moisture binder, and high viscosity type CMC acts as a gelation agent [120].

Furthermore, it is used in semisolid dairy products, salad dressing, and fruit syrup as a thickener [183,184,185]. Nowadays, in fruit syrup or juice, CMC is used as a hydrogel (dewatering agent) to ensure food safety. In the conventional thickening process, heat is essential, where the super absorbing or dewatering process in the presence of heat is not essential [126]. CMC is utilized in milk and cream products, condiments and bakery food, acidic dairy products, and ice cream, acid beverages, spreads, and wine as an emulsifier [186,187]. In ice cream, 0.5% CMC is used as a stabilizer to produce the best quality ice cream. CMC with medium viscosity enhances the texture quality and provides creamy mouthfeel via eliminating the over-crystal growth of lactose in cream [120,185]. In cocoa and acid milk drinks, CMC is often used for stabilizing the texture of drinks, preventing sedimentation and layer forming in drinks at low and high temperatures [130]. Due to stabilized dietary fiber or nutrients in fruit-based beverages, CMC has recently been used as a stabilizer in a drink as a blended form of CMC and gum tragacanth [188].

In the last couple of years, the use of CMCs in food has moved from a normal to advanced level in food as they are hygienic, biocompatible, and human disease prevention or dietary management. For example, due to the prevention of gastrointestinal infection, Dafe et al. (2017) [189] developed CMC/k-carrageenan mixed food vehicle to supply probiotic-based food in the colon. While suggested, food controlled the gastrointestinal tract or mucosa’s health and improved the immune system. Similarly, in 2019, Ngamekaue et al. [190] proposed another coating material for delivering a herbal oil-loaded microcapsule (a blend of holy basil oil and gelatin) the intestine. Simultaneously, a CMC/beeswax coating composite protects the microcapsule from acid, moisture, or oxidative elements and helps control the herbal product after reaching the intestine. Suggested herbal products act as good antioxidants, anticancer agents, etc., in the human body and help protect non-communicable diseases. Therefore, due to extending the shelf life, essential oil and grape seed extract bio-based CMC coatings have also been applied on seafood [191].

High fats in the food (meat) are a significant barrier to making healthy food products. To solve this problem, CMC derivatives are used as fat replacers or reducers. As fat replacers, Gibis and colleagues (2015) [192] used CMC and MCC (microcrystalline cellulose) when making fried beef patties where 0.5% CMC concentration was chosen as a good fat replacer and excellent flavor, texture, and juiciness supplier. On the other hand, Han et al. (2017) [193] designed high nutrition and lowest fat-based healthier meat products. During meat processing, CMC and other dietary fibers (such as cellulose, chitosan, inulin, pectin, etc.) are used as fat reducers, nutrition enhancers, and texture modifiers that help to reduce the chances of colon cancer, cardiovascular disease, etc.

In the last decade, some researchers have disclosed the use of CMC as food packaging material. Khezrian et al. (2017) [194] developed an essential oil doped montmorillonite/chitosan/CMC nanocomposite-based active packaging material to extend the shelf life of camel meat. Moreover, as a primary packaging material, biodegradable and ecofriendly PVP–CMC hydrogel film is also widely used for food goods [195]. CMC/PVA/zeolite doped with a metal cation-based film has been reported as a biodegradable and antimicrobial packaging material. This antimicrobial property prevents food from spoiling and increases the shelf life of goods [196]. CMC-CHPS (chickpea hull polysaccharides) films produce antioxidant and antimicrobial activities in the packaging materials [197]. Recently, high thermal stability and high stiffness-based packaging materials have also been developed by conjugation of CMC, dopamine, and montmorillonite [198].

### 4.3. Application in the Water Treatment Process

In the present day, water pollution has become one of the most crucial issues worldwide. An immense number of pollutants come from various industries and household deeds every day and enter into aquatic environments, which further causes several types of disorders in different living organisms and human beings. There is no absolute limit on how many types of pollutants can be present in wastewater that has come from multiple industries or other household activities. However, to the best of the author’s knowledge, CMC-based materials have been mainly used thus far for the removal of various organic-inorganic dyes [74,199,200,201], inorganic ionic pollutants (both anions and cations) [202,203,204,205], as well as various radioactive species [83,206,207,208] from polluted waters in different experimental conditions.

Dyes are considered the most hazardous compounds in aquatic environments [209,210,211,212]. A wide spectrum of their applications in various industries such as food, paint, textiles, pulp, paper, rubber, plastics, tannery, cosmetics, and dozens of structural and manufacturing industries have turned the waste dye effluents into a crucial factor in wastewater treatment works [213,214,215] Moreover, their long-term durability against light, heat, and other oxidizing agents, slow rate of biodegradation, and variety of chemical compositions have made them a more intricate issue in environmental pollutions [216]. Furthermore, inorganic ionic pollutants, especially heavy metal ions, and some reactive anions have been marked as highly harmful to the health of various living beings [217,218,219,220].

CMC has been demonstrated as active material in various water treatment works during the past few years by many researchers. However, some researchers have elicited some excellent innovative hybrid materials with CMC to remove various pollutants from wastewater in recent years, as presented in the Supplementary Materials (Table S4). For example, Salama et al. (2018) [201] synthesized a nano-adsorbent material (i.e., CMC/Fe_3_O_4_) by the co-precipitation method for the adsorption of methylene blue dye from the experimental polluted aqueous solution, where about 48 mg of methylene blue hues were adsorbed per gram of the adsorbent at pH 3, and maximum adsorption efficiency was obtained at pH 7 (i.e., 64 mg/g). In the same year, Hong and his co-workers (2018) [70] investigated the scavenging performance of another CMC embedded polyurethane composite against various metal pollutants from industrial waters. The composite material demonstrated some excellent adsorption efficiencies against various ionic contaminants. Up to 216.1 mg of Pb^2+^, 78.7 mg of Cu^2+^, and 98 mg of Cd^2+^ ions were removed by each gram of the adsorbent. Most recently, Manzoor and his co-workers (2019) [221] synthesized a chitosan/CMC hybrid adsorbents using arginine cross-linkers that showed a better adsorption capacity against Cd^2+^ and Pb^2+^ ions (up to 168.5 mg/g and 182.5 mg/g, respectively) in experimental conditions. Gasemloo et al. (2019) [222] demonstrated a sulfated-CMC-based nano-filter membrane technology (cross-linked with glutaraldehyde) that showed a high removal efficiency against Cr(VI) at optimum conditions (i.e., maximum pollutant removal 79.85% at 3 bar pressure and pH 4). Additionally, Wei et al. (2015) [223] demonstrated an ultrathin fibrous CMC-based cross-linked nanocomposite material (cross-linked with epichlorohydrin) that showed a super-dynamic removal of Cd^2+^ ions from experimental wastewater. Moreover, up to 150.60 ± 10.47 mg of the pollutants were removed per gram of the adsorbent at optimum conditions. Likewise, other researchers have also reported dozens of such effective outcomes that reveal the potentialities of CMC-based composite materials for efficient removal of various organic-inorganic pollutants in wastewater treatments.

Moreover, some researchers have reported some excellent adsorption capacity of CMC-based composite materials against radioactive pollutants from wastewaters. For instance, ^137^Cs (half-life 30.1 years) is a randomly found radioactive element in nuclear wastewaters, which is considered as causing many diseases such as genetic disordering, cancer, etc. [224,225]. In 2018, Rethinasabapathy et al. [207] synthesized a layered structured multifunctional polyhedral oligomeric silsesquioxane (POSS) modified Fe-aminoclay/CMC composite that exhibited adsorption capacity against radioactive Cs ions as well as methylene blue and chrysoidine dyes (152 mg/g, 438 mg/g, and 791 mg/g, respectively). In the following year, Zhang and his co-workers (2020) [226] fabricated a CMC-based hybrid adsorbent (cross-linked with ammonium phosphomolybdate). They demonstrated its excellent adsorption capacity against radioactive ^137^Cs (64.20 mg/g) from nuclear wastewater. Furthermore, Eun et al. (2020) [179] demonstrated a Prussian-blue embedded CMC nanofibrous membrane technology that showed a competitive adsorption capacity against radioactive ^137^Cs (maximum 130 mg/g) from laboratory-based experimental aqueous environments. A few years ago, Shao et al. (2008) [206] synthesized a CMC grafted multi-walled carbon nanotube (CMC-g-MCNT) adsorbent for radioactive uranium sorption from nuclear wastewater. Uranium is an essential element of nuclear power plants, and atomic energy programs have found UO_2_^2+^ soluble cationic forms in wastewater around the plants [206]. The authors demonstrated an extreme adsorption capacity of their synthesized CMC-g-MCNT sorbent against UO_2_^2+^ nuclides (i.e., up to 98% of pollutants were removed using only 1g/L adsorbent). Furthermore, Cai, along with his research team (2017) [83], fabricated a core-shell structured Fe_3_O_4_-modified CMC composite material that exhibited a moderate extraction capacity against the radioactive ^152+154^Eu nuclide (i.e., maximum removal 2.78 × 10^−4^ mol/g at 293 k) from wastewater.

The above-mentioned discussion was just a glimpse of the potentialities of various CMC-based composite materials for wastewater treatment purposes. More effective research outcomes with a brief detail have been summarized in the Supplementary Materials (Table S4). Here, it is worth mentioning that the CMC-based composites will naturally exhibit a better adsorption tendency toward the metal ions and other cationic pollutants than anionic contaminants due to the presence of electronegative carboxymethyl groups (^-^COOH) on their surface. Electrostatic repulsion between the anionic pollutants and the opposing surface charges makes it harder for the adsorbents to bind the negatively charged impurities efficiently for a long time. Perhaps this is the only reason behind the frugality of research attempts in the literature about anionic pollutant removal by CMC-based composite materials in wastewater treatment compared to cationic pollutants.

Therefore, we suggest focusing on the fabrication of CMC-based, more effective, and low-cost hybrid materials, especially nano-hybrid super-absorbent materials. Future research works to remove various cationic pollutants such as metal ions, cationic dyes, and different cationic radioactive materials in wastewater treatment are needed. Additionally, surface modification of the CMC-based adsorbents by hybridization with varying types of organic-inorganic positively charged species may make them more promising in the future for the treatment of both cationic and anionic pollutants contemporaneously.

### 4.4. Biomedical Application

Due to the characteristic surface properties for facile cell adhesion, low cytotoxicity, excellent biocompatibility, biodegradability, and cell viability, CMC and CMC-based hybrid materials have found a wide range of applications in biomedical fields during the past few decades. In the literature, a series of research evidence on CMC-based biomaterials have been reported so far in tissue engineering, wound dressing, bio-sensing, bio-imaging, bone regeneration, drug delivery, and likewise dozens of working fields in biomedical technology. However, all of these fields are interrelated with each other. For convenience, the application fields are separately discussed below.

#### 4.4.1. 3D Bioprinting Process

**Figure 5:**
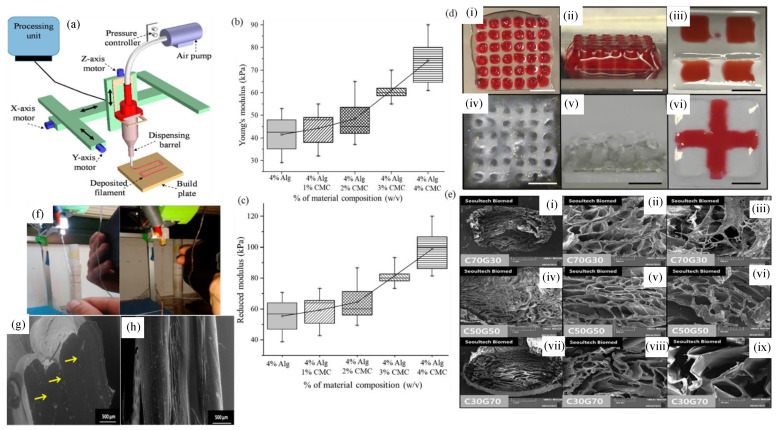
(**a**) The schematic diagram of in-house built 3D bio-printer [230]; (**b**,**c**) Young’s and Reduced Modulus of alginate/CMC hydrogels (In the case of alginate/CMC, the shear-thinning effect is more pronounced, reflecting the better possibility of this hybrid material bio-print the clinically relevant scaffolds [230]; (**d**) Pictures show multi-material printing of thick (>3 mm in height) scaffolds. (**i**,**ii**) Top (**i**) and side (**ii**) views of NorCMC (1:2) scaffold printed with Pluronic (red). (**iii**,**iv**) Scaffold after Pluronic is dissolved in PBS. (**v**,**vi**) NorCMC (1:2) scaffolds printed with fast degrading cCMC (1:4) (red). Scale bars are 5 mm. Reproduced with permission from [235]. Copyright 2021, Elsevier; (**e**) SEM images of the dried CMC-GC gels with different compositions. Reproduced with permission from [208]. Copyright 2021, Elsevier; (**f**) Photos of the neat PDMS and composite Na–CMC/PDMS filaments used in FDM; SEM images, at different magnification [233]; (**g**,**h**) the composite filament printed in layers. Arrows indicate the interface between different layers [233].

Recently, CMC-based biomaterials are becoming popular in the 3D bioprinting process, which is a remarkable technology in tissue engineering. In this process, living tissue scaffolds are reproduced by layer-by-layer deposition of an appropriate biomaterial using a computer-controlled 3D printing system [227,228,229]. Habib and his co-workers [230] synthesized a hybrid hydrogel using CMC as a potential biomaterial for the 3D bioprinting process (Figure 5a). CMC was hybridized with sodium alginate, another well-developed biocompatible material for this process. However, the incorporation of CMC with alginate materials enhanced its solution viscosity and improved its printability (Figure 5b,c). In some previous studies, alginate-CMC hybrid hydrogels have also been used in manufacturing beads for drug delivery purposes [231,232]. In a recent survey, Calcagnile and his research fellows (2018) [233] reported a low-cost fused deposition model for 3D-printing of the human heart by using poly-(dimethylsiloxane)/Na-CMC composites. Na-CMC moieties were used to mimic the soil’s slimy effect for the composite and improve its tactile properties (Figure 5f–h). Most recently, Janarthanan et al. (2020) [234] introduced a novel co-polymeric hydrogel of hyaluronic acid/CMC (cross-linked with N-acyl-hydrazone) that exhibited super post-printing stability without any supporting materials. Its exceptional elastic properties (e.g., fast recovery ever after 50% strain) have made it more promising for soft-tissue engineering applications. Moreover, the characteristic self-healing and shear-thinning properties of the material allowed them to print different shapes of the 3D architecture for this purpose. The same year, Ji et al. (2020) [235] presented two novel CMC-based macromers (modified with amide and ester cross-linker, respectively) as cost-efficient bio-inkers for printing differential 3D-architectures with increased complexity (Figure 5d). In another study, Janarthanan et al. (2020) [208] synthesized three different types of CMC/glycol chitosan-based hydrogels via simple in situ–ionic interactions and gelling Schiff’s base reaction (Figure 5e). The biocompatible hydrogels (biocompatibility was evaluated using MC3T3 mouse osteoblast cells) showed enhanced stability after their in vitro 3D printing. Moreover, the 3D-printed gels showed their potentialities in sustained-release drugs (i.e., lactoferrin) up to 21 days at optimum pH conditions. Melili et al. (2020) [236] reported a photocurable bio-ink based on methacrylate-CMC that showed optimum potentialities to produce 3D-shaped hydrogels with good mechanical properties and swelling behavior. However, in that study, the authors revealed the applicability of CMC-based biomaterials in the bio-printing process via the digital light processing (DLP) method. Likewise, many more works have been reported in the literature that proves the feasibility and importance of CMC-based biomaterials in this context. As an emerging field in tissue engineering, this process demands many more future works from researchers based on CMC-based biomaterials.

#### 4.4.2. Drug Delivery

**Figure 6:**
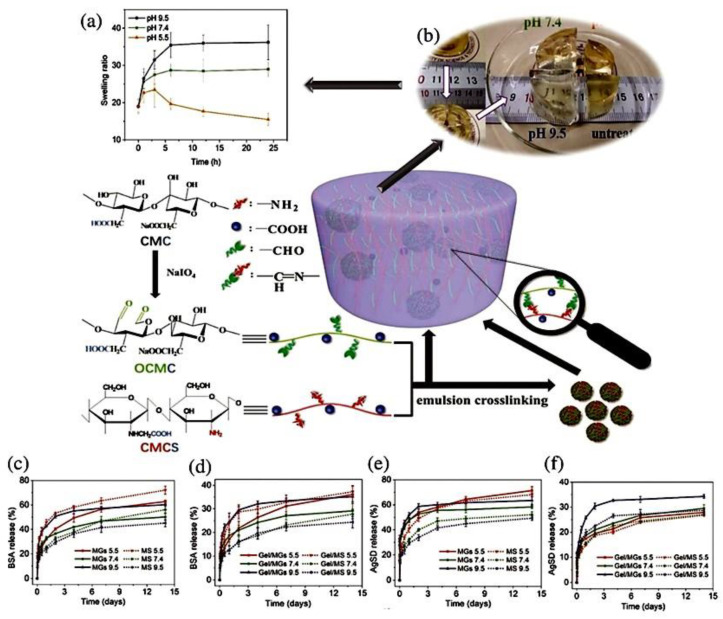
(**a**) Swelling ratio curve of Gel/MGs with 30 mg/mL, MGs were incubating in different pH. (**b**) Picture of Gel/MGs after incubating in different pH for six h. Values reported are an average *n* = 5, ± standard deviation. (**c**,**d**) The BSA release profile of MGs, MS, Gel/MGs, Gel/MS in different pH. (**e**,**f**) AgSD release profile of MGs, MS, Gel/MGs, Gel/MS in different pH. Values reported are an average *n* = 5, ± standard deviation. Reproduced with permission from [240]. Copyright 2021, John Wiley and Sons.

CMC-based composite materials are also widely used in the drug delivery process. Many researchers have reported the delivery of potent pharmaceutically active compounds through CMC-based biomaterials. For instance, Oliveira et al. (2017) [237] developed a PVA/Na-CMC hydrogel that presented an adequate release of flavonoids and phenols to the wound sites aside from excellent elasticity and swelling property with good antimicrobial properties. In the previous year, Namazi et al. (2016) [238] developed a CMC/MCM-41 (Mobile Composition of Matter no. 41) nanocomposite hydrogel material as a potential drug carrier to wound sites. Agarwal et al. (2013) [239] developed an artificial membrane of PVA/poly-(ethylene oxide)/CMC by the freeze-drying and solvent-casting method for drug delivery purposes. Ciprofloxacin hydrochloride, an anti-bacterial agent, was incorporated into the highly porous matrix of the membrane, and the capability of the matrix for drug delivery was tested. A controlled drug release of the matrix for up to 10 h was observed, proving it as a potential biomaterial for wound dressing purposes. Du et al. (2019) [240] fabricated a co-polymeric microgel based on carboxymethyl chitosan and oxidized CMC loaded with two protein drugs, bovine serum albumin, and silver sulfadiazine. Later on, they embedded the drug-loaded microgel into a hydrogel via Schiff base reaction. The most contrasting aspect of the report was the pH-sensitive drug delivery system of the hydrogels with an excellent antibacterial activity (tested with *S. aureus*) (Figure 6). Joorabloo et al. (2019) [241] fabricated a biocompatible and non-toxic nano-ZnO/PVA/CMC hydrogel that showed good cell viability with an enhanced antibacterial activity (tested against *S. aureus* and *E. coli* bacteria).

Moreover, the addition of heparin in the gel structure improved the matrix in vitro wound healing ability. The gel structure can be used as a potential carrier of the pharmaceutically active substance in the wound healing process.

Furthermore, cellulose-based microcarriers are well-known as biocompatible support for cell attachment, augmentation, and proliferation. However, such cellular functions (i.e., adhesion, proliferation, etc.) are highly facilitated when microcarrier surfaces are modified with various anionic or cationic species instead of the neat-cellulose carriers. Recently, Ramezani Kalmer and his co-workers (2019) [242] synthesized a carboxymethylated multifunctional water-soluble cellulosic microcarrier (average diameter 1650 ± 100 µm, size 1500–1800 µm) from diethylamonioethyl cellulose (DEAEC) with the contemporaneous presence of anionic and cationic functional groups. The CMC/DEAEC carriers demonstrated themselves as a high-performance supporting biomaterial for cell adhesion, immobilization, and proliferation. Cheng et al. (2018) [243] fabricated a core-shell structured microcapsule carrier for cells or various therapeutic agents by the conventional centrifugal microfluidic system and electrostatic spraying. The Na-alginate solution was used as the shell fluid in the device structure, whereas the Na-CMC solution was used as the inner-core fluid for the full delivery of drugs or cell culture. Kandalam et al. (2020) [244] used Na-CMC as a potential vehicle for pharmacologically active microcarriers containing stem cells from the apical papilla and brain-derived neurotrophic factors, which was proposed as a possible therapy for spinal cord injury. Ahmadi et al. (2011) [245] used a diluted solution (2.3% *w*/*v*) of Na-CMC to enhance the cell viability of their fabricated micro-spherical polylactide-co-glycolide carrier.

It is worth noting that microcarriers have gained copious interest over the past years for drug delivery, cell transplantation, cell expansion, and tissue bulking in a minimally invasive manner. However, in the literature, the volume of research works based on CMC-based microcarriers is comparatively lower than that of the other biomaterials. Therefore, there is still a vast scope of research in this field that may bring more advanced and potential CMC-based biomaterials into the limelight in this context.

#### 4.4.3. Tissue Engineering

##### Wound Dressing

CMC is widely used to improve the characteristics and effectiveness of various wound dressing and wound healing materials in various clinical applications due to their excellent binding capacity to the internal body cells, biocompatibility (i.e., with skin, bone, and mucous membranes), and strong hydrophilic property (due to the presence of anionic carboxylic and hydroxyl groups in internal network structure and surface as well) [246,247]. Furthermore, blending capability with various water-soluble organic polymers such as poly-(vinyl alcohol) (PVA), poly-(ethylene glycol), etc., have widened the application field of CMC-based biomaterials in wound dressing purposes [248,249]. In this regard, numerous structures of such materials have been used. Amongst them, CMC-based hydrogels [250,251,252], films [253,254], fibers [255,256,257], wafers [258,259,260], gauzes [261,262], and nanoparticles [263,264,265] have gained much interest during the past decades.

Hydrogels are three-dimensional (3D) porous structured materials formed by regular cross-linking of various identical polymeric materials. They are hydrophilic and are capable of holding a high amount of water in their internal porous networks [266,267]. Their tissue-like structure and such high water absorbing and retaining properties have encouraged researchers to use them in potent drug delivery and wound dressing applications [268]. CMC-based hydrogels have gained much interest in this context mainly due to their excellent capability of maintaining the moist environment around the targeted wound area that accelerates the cell growth, facilitates the functioning of enzymes and hormones, and overall, enhances the cell growth factors significantly [269,270]. Additionally, they promote the proliferation and migration of various keratinocytes and fibroblasts that reduce the wound healing time and decrease the formation of scars [271,272,273]. A few years ago, Capanema et al. (2017) [271] developed a poly-(ethylene glycol) modified CMC-based hydrogel with flexible swelling behavior and excellent mechanical properties. The degree of swelling exhibited an extensive range (i.e., from 100% to 5000%), depending upon the cross-linking of the gels and aspect ratio of the poly-(ethylene glycol) DS value of the used CMC.

Moreover, excellent cell viability (i.e., 95% viability responses) of the synthesized gels with its non-cytotoxic nature and sponge-like structure proved it to be a potential material for wound dressing. In recent work, Bayindir bilgic and co-workers (2019) [274] synthesized a co-polymeric hydrogel material of chitosan and CMC loaded with alpha-tocopherol and reported its excellent wound healing performance without inducing any cytotoxic effects. The cell proliferation analysis showed excellent cell viability of the synthesized hydrogel with the 3-(4,5-dimethyl-2-thiazolyl)-diphenyl tetrazolium bromide (MTT) method. Joorabloo et al. (2019) [241] synthesized a nano-ZnO/PVA/CMC-based nano-biocomposite hydrogel material for wound dressing via a freeze-thawing form. The hydrogels showed excellent biocompatibility, cell viability, and mechanical properties with controllable water vapor transmission rate and degree of swelling that facilitated wound healing, aside from just protecting the wound surface. Most recently, Koneru et al. (2020) [44] fabricated a Na-CMC/hydroxypropyl methylcellulose hydrogel for potential drug delivery and wound healing purposes.

Furthermore, they incorporated the extract of grapefruit seeds into the hydrogel matrix and showed enhanced antibacterial activity of it as a consequence. The field emission scanning electron microscopy and the field emission transmission electron microscopic analysis revealed that the grapefruit seed extracts’ glycerides, while combined with the Na-CMC of the matrix, some micelles were formed, which was responsible for the enhanced antibacterial performance. However, an innovative idea of utilizing the naturally extracted herbal components with CMC-based biomaterials for faster wound healing performance was introduced in that study. Likewise, numerous research works have been reported in the literature that has proved the feasibility and potentialities of CMC-based hydrogels for wound dressing and wound healing applications [42,275,276,277,278,279].

Significant challenges in fabricating ideal wound dressing materials are attaining suitable pore sizes of the materials for the identical surface of the wound sites, obtaining an excellent mechanical property, appropriate water-vapor transmittance, and compatibility with the wound tissue sites via a facile preparation technology. Recently, Li et al. (2019) [280] reported a bi-layer wound dressing material composed of PVA/CMC/poly-(ethylene glycol) that showed comparatively higher resistance against bacterial penetration with an excellent mechanical strength than that of the single-layered hydrogels. They synthesized the material using a facile one-step thawing-freezing method (i.e., simple phase separation process) without incorporating any kinds of excessive chemical additives that could further induce toxicity for wound skins after dressing. Non-toxicity of the hydrogel was demonstrated by the cytotoxicity test based on the L929-fibroblast cell. Furthermore, pore-sizes of the hydrogels were proven to be flexible and could be tailored by varying the aspect ratio and concentration of the PVA in the hydrogel. In the same year, another research group of Jantrawut et al. (2019) [248] developed a low methoxyl pectin/gelatin/CMC-based bi-layered hydrogel film that also exhibited a high fluid uptake capability and water retention capacity with a high integrity value. Additionally, well-controlled drug release of the hydrogel films loaded with povidone-iodine demonstrated its potentialities to be used as an alternative vehicle for the delivery of aseptic and/or antibiotics to the moist wound sites to accelerate wound healing without any kinds of posterior bacterial infections. Most recently, Sharma and his co-workers (2020) [281] fabricated a photosensitizer embedded in a sodium alginate/pectin/CMC bi-layered film for antimicrobial photodynamic therapy (APDT) of infected wounds. This process is used to treat injuries that have been infected by antibiotic-resistant bacteria. The most critical part of the entire process is the delivery of photosensitizing material at an appropriate concentration into the infected site. This problem was smoothly resolved by the fabricated SA/PC/CMC bi-layered films in this study.

Moreover, other than the hydrogel networks, CMC-based fibers and gauzes are also widely used for wound care. The fibrous structure of the wound dressing material provides some extra advantages such as size flexibility, high fluid absorbency, and non-adherence to the wounds. Additionally, they can be easily cut into a particular size according to the extent of the injury and possess a comparatively lower rate of infection proliferation at the surroundings of the wound site. In ancient research work, Waring et al. (2001) [282] fabricated a novel Na-CMC fiber structure that demonstrated its moderate potential for fluid immobilization by gel-blocking, which has inspired further research attempts to develop more advanced fibrous materials using CMC. Similarly, Zhao et al. (2015) [283] fabricated carboxymethyl cotton knitted fabrics using various solvents such as water, isopropanol/water, and ethanol/water mixtures at specific aspect ratios. Doh et al. (2013) [284] developed a nonwoven composite of CMC and PE/PP bi-component fibers by a wet-laid process that exhibited all of the critical properties used in an advanced wound dressing material. Li et al. (2016) [278] synthesized a hydrogel fiber of gelatin/poly-(ethylene glycol) composite by a gel-spinning method where dialdehyde-CMC (DCMC) was used as a cross-linking agent. However, the DCMC composite is a well-known and widely used cross-linking reagent for polymeric networks propounded by many other researchers in the literature to fabricate different wound care materials [82,285,286,287].

##### CMC in 3D Scaffold Materials

CMC-based biomaterials are widely used in tissue engineering applications. During the past few years, three dimensional (3D) porous scaffolds of numerous biopolymers such as collagen [288,289,290,291], chitosan [292,293,294], bacterial cellulose [295], sodium alginate [296] as well as other various CMC-composites [297] have been used to mimic extracellular polymeric substances in tissue engineering. However, their cell viability for in vitro applications, cell adhering capacity, and toxicity are some promising challenges in this context. Recently, Gheysari et al. (2019) [298] fabricated a highly porous hydroxyapatite-gel/CMC nanocomposite scaffold by a facile lyophilization method that exhibited high cell adhering capacity, non-toxicity, and good cell viability with a high water uptake capacity (up to >600%). Moreover, the synthesized scaffolds were biodegradable with satisfactory cell viability (i.e., >80% even after 48 h), revealed by in vitro experimental incubations. In the same process, Al-Abboodi and his team (2014) [299] developed a novel hydroxyphenylpropionic acid/CMC-tyramine hydrogel with moderate porosity, excellent biocompatibility (with COS-7 cells), and similar mechanical properties with soft body tissues and organs. Furthermore, the 3D scaffold showed its tuneable mechanical properties, which proved to be highly promising in applications in patients’ bodies of any age and dietary intakes for their cursorial recovery. Ninan et al. (2013) [300] fabricated a highly porous novel scaffold of CMC/pectin/micro fibrillated cellulose for potential tissue regeneration. Cell viability test with NIH3T3 fibroblast cell lines proved its high potential to be used as an ideal polymeric matrix for such an application. Previously, Jiang et al. (2008) [301] fabricated a porous scaffold with cheaper materials such as CMC, chitosan, and nano-hydroxyapatite with excellent bioactivity, biodegradability, and compressive strength (i.e., up to 3.54 MPa) in the same process. Rodrigues et al. (2013) [302] developed a CMC-based scaffold using adipose-derived mesenchymal stem cells to repair skin lesions and extensive burns. Moreover, other than the conventional lyophilization method, Chen et al. (2007) [46] prepared a chitosan/CMC polyelectrolyte complex 3D scaffold for pulp tissue engineering by a facile freeze-drying process. Recently, in the same process, Kanimozhi et al. (2018) [303] fabricated a chitosan/PVA/CMC scaffold as a potential biomimetic material for likely soft tissue engineering. However, the authors ensured another conventional method (salt-leaching process) to fabricate scaffolds that outstripped the previous scaffold materials in terms of antibacterial activity, mechanical strength, and cell viability.

##### Bone-Tissue Engineering

Many researchers have evolved a new interest in bone tissue engineering for various clinical applications. For the replacement of synthetic bones, numerous graft polymeric materials have gained much interest in the past few years that have promoted bone regeneration technology during spinal, orthopedic, and dental surgeries [304]. To date, a tremendous amount of research evidence has supported the feasibility of the application of CMC-based biomaterials for bone tissue engineering and bone regeneration purposes. For instance, Aoshima and Jo (2013) [305] demonstrated an in vitro stimulation of CMC compounds for adhesion and proliferation of mouse fibroblasts. Clarke and his co-workers (2007) [306] reported that the presence of CMC in calcium phosphate (β-TCP) granules enhanced the activity of alkaline phosphate (ALP) and proliferation of stromal cells in the human bone marrow.

Furthermore, a significant reduction in murine bone marrow progenitors induced osteoclastogenesis by CMC has also been reported in another study [307]. Recently, Qi et al. (2018) [304] demonstrated an effective in vivo bone regeneration in a mouse calvarial defect model and in vitro osteoblast differentiation of human mesenchymal stromal cells by using a non-woven CMC sheet (loaded with calcium phosphate). Most recently, Namkaew et al. (2021) [308] reported a polyvinyl alcohol-based scaffold material for supporting cartilage formations at post-surgical conditions. The incorporation of CMC with this porous scaffold material improved the physical and swelling properties that made it more suitable for this purpose. Sharmila et al. (2020) [39] reported a plant-based scaffold composite from the extracts of *Spinacae olareacea* medicinal plants with CMC and synthetic alginate (i.e., Alginate/CMC/SO) for bone tissue engineering. The scaffold exhibited excellent cell viability and good biocompatibility revealed by in vitro tests with MG63 human osteosarcoma cells. In another recent work, Manjubala and her research fellows (2018) [309] developed a CMC/hydroxyapatite-based composite scaffold mimic of natural bones. Hasan et al. (2018) [310] synthesized a scaffold of chitosan/CMC/cellulose nanowhiskers (decorated with silver nano-particles) to overcome bone-related complexities like osteomyelitis. The scaffolds exhibited an enhanced antibacterial activity, negligible cytotoxicity (assayed with MG63 cells), and good cell viability with improved mechanical properties. A few years back, in 2015, Saintya et al. [311] synthesized a cytocompatible, highly stable scaffold of chitosan/CMC/mesoporous-wollastonite composite particles as a potential biomaterial for bone tissue engineering. In the previous year, Chen et al. (2014) [312] fabricated a synthetic scaffold of a bioactive glass/chitosan/CMC composite that effectively functioned as a hemostatic agent in orthopedic osteotomy (i.e., bone cutting process) as a better replacement of traditional bone-waxes. Likewise, Liuyun et al. (2009) [313] synthesized a nano-hydroxyapatite/chitosan/CMC-based scaffold that showed all of the critical properties such as structural stability non-cytotoxicity, good cell-viability, tissue biocompatibility with good mechanical properties that proved it to be another potential material for bone tissue engineering. Recently, Priya et al. (2021) [314] reported a biocompatible CMC scaffold material cross-linked with citric acid for potential bone-tissue engineering applications. Likewise, many scaffold composites have been reported in the literature based on CMC in this context. However, efforts of synthesizing new materials have never been paused. Fabrication of new biomaterials with improved physicochemical properties in a more cost-efficient way may help to develop this field further in the future.

#### 4.4.4. Bio-Sensing and Bio-Imaging

**Figure 7:**
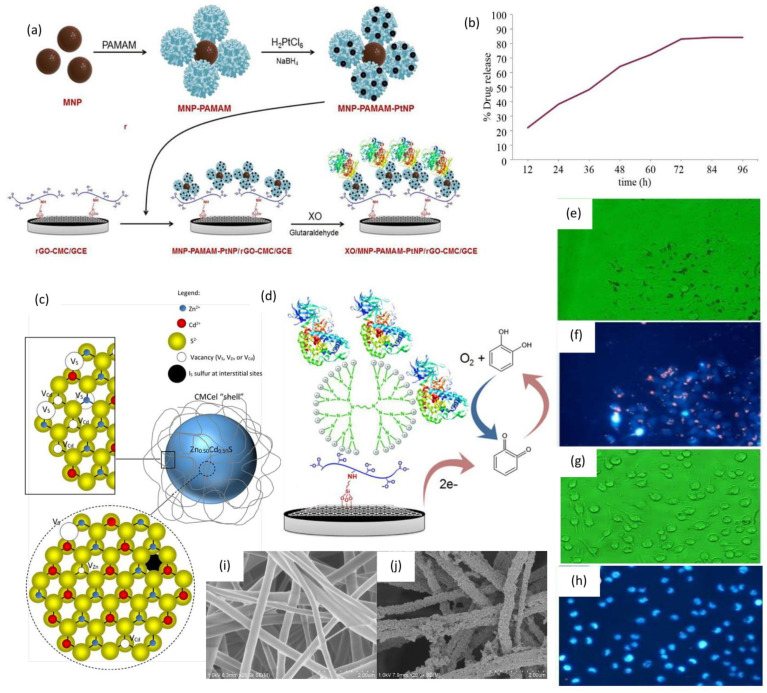
Application of CMC in bio-sensing and bio-imaging (**a**); Schematic display of the preparation of the MNP-PAMAM-PtNP and the XO/MNP-PAMAM-PtNP/rGO-CMC/GCE enzyme electrode. Reproduced with permission from [319]. Copyright 2016, Elsevier; (**b**) Drug release profile from the multifunctional nanoparticles [329]; (**c**) Schematic representation of Zn_0.50_Cd_0.50_S quantum dots stabilized by CMC polymer (not to scale). Reproduced with permission from [327]. Copyright 2018, Elsevier; (**d**) The mechanism for Tyr-catalyzed electrochemical detection of catechol. Reproduced with permission from [320]. Copyright 2015, John Wiley and Sons; (**e**) phase contrast image; (**f**) florescent image of MCF7 cells (**g**) phase contrast image; (**h**) florescent image of L929 cells with folate conjugated CMC with QDs [329]; SEM images of CMC/cellulose nanofibers (**i**), and PANI/CMC/cellulose nanofibers (**j**). Reproduced with permission from [316]. Copyright 2015, Elsevier.

Besides tissue engineering and wound caring applications, CMC and CMC-based hybrid materials are also used as biosensors for detecting the presence of various biogenic compounds in the human body or other living organisms. In an ancient study, Wu et al. (2004) [315] fabricated an optical glucose biosensor by entrapping an enzyme (i.e., glucose oxidase) into a xerogel matrix of tetraethyl-orthosilicate and hydroxyethyl CMC polymers. The biosensor exhibited an excellent analytical feature to detect the presence and amount of glucose in urine samples. A few years back, in 2015, Fu et al. [316] synthesized hierarchical structured polyaniline/CMC/cellulose nanofibers to determine the presence of catechol through the laccase biosensor (Figure 7i,j). It is worth mentioning that laccase biosensors are commonly used to detect and monitor various phenolic compounds [317,318]. However, the authors in this study showed that the as-prepared laccase/polyaniline/CMC/cellulose/glassy carbon electrode structure demonstrated better detection quality toward catechol than the other laccase-based biosensors with high reproducibility and repeatability. In the following year, Borisova et al. (2016) [319] developed a layer-by-layer biosensor of reduced graphene oxide/CMC/platinum nanoparticles (decorated with polyamidoamine G-4 dendrimers and modified magnetic nanoparticles) for the detection of xanthine in fish samples (Figure 7a). In another research work, the same research group fabricated a nanostructured electrochemical biosensor using polyamidoamine G-4 and CMC-modified graphene oxide (Figure 7d) [320]. This time, the biosensor exhibited a susceptible analytical performance (i.e., 6.3 A/M), even at a low detection limit (0.9 nM).

In the past decade, Villalonga and his research fellows (2007) [321] developed an amphoteric biosensor for xanthine detection and quantification β-cyclodextrin-branched CMC was used to modify and immobilize xanthine oxidase enzyme on the electrodes of an electrochemical bio-sensing system. Recently, Cui et al. (2019) [322] used a reduced graphene oxide/CMC blend to modify the glassy carbon electrodes of an electrochemical biosensor to detect vitamin B_6_. Kocabay et al. (2012) [323] developed a novel CMC/gelatin/superoxide dismutase-based biosensor to see the number of superoxide radicals (O_2_·^-^), which is considered as the primary species of ROS (i.e., reactive oxygen species) that can bring various types of reversible or permanent damages to several biomolecules. The biosensor exhibited high accuracy (e.g., correlation coefficient, R^2^ = 0.9994) and high sensitivity toward the targeted species, even at a low detection limit (i.e., 1.25 × 10^−3^ mM). Moreover, almost 90% sensitivity of the biosensor remained unchanged even after 80 days of its continuous usage. A contrasting aspect of these CMC-based biosensors is that they are susceptible to their targeted compounds and provide accurate measurements. Some researchers directly attributed the CMC-based moieties of these materials to attain such accuracy and sensitivity [324,325,326]. Therefore, CMC-based biomaterials have great potential in preparing thousands of effective biosensors in the future if proper research attention is provided in this field.

Furthermore, various CMC-based composites have also been propounded as potential materials for in vitro bio-imaging purposes. For instance, Mansur et al. (2017) [327] synthesized a novel quantum dot/CMC nano-conjugate material that showed its high potentialities for use as a fluorescent nanoprobe for in vitro bio-imaging of living cells (Figure 7c). In the same year, the authors developed a multifunctional, composition-tuneable semiconductor nanocrystal of a Zn_x_Cd_(1-x)_S quantum dot/CMC composite that exhibited its effectivity as a fluorophore for the bioimaging of living cells (HEK293T cells were used for the bioimaging test) [328]. Aswathy et al. (2012) [329] synthesized a multifunctional biocompatible nanoparticle based on CMC for specific recognition of cancer cells (Figure 7e–h). Sivakumar et al. (2013) [330] fabricated a CMC/magnetic nanoparticle system where CMC-moieties were used as a surface coating agent. The nano-bio materials contemporaneously acted as a vector for targeting cancer cells, their imaging, and finally, the efficient delivery of therapeutic agents to the targeted cells.

However, the precedents mentioned above are just a glimpse of the application strategies for CMC in biomedical fields. Likewise, many research efforts on CMC-based biomaterials have been reported so far in the literature. However, further research is still required to develop this field by introducing advanced materials in the existing technologies and developing newer promising technologies to facilitate clinical, surgical, and medical applications.

### 4.5. Application of CMC in Pharmaceutical Industries

Some naturally extracted compounds have gained much interest in the nutraceutical and pharmaceutical industries in recent years. For instance, red palm oil, an essential oil with various health benefits and pharmaceutical interests, CMC, antioxidant compounds, etc., are being used frequently. Among them, CMC and its derivatives (composite/copolymer) have received ample attention for use in pharmaceutical products due to its properties such as biocompatibility, long half-life in vivo, high stability and drug binding capacity, pH-sensitivity (for the presence of carboxy group [331]), and best reliable carrier, etc. [332,333].

CMC plays various pharmaceutical applications as binders, stabilizers, emulsifiers, film-forming components, reliable carriers, etc. These applications greatly depend on the purity, DS value, solubility, particle size, etc., of the synthesized CMC and their derivatives. Virtue is soundly attached to pharmaceutical products, where it acts as an essential factor in the drug. It is commonly said that CMC requires high purity for pharmaceutical or food products, and this is defined by the presence of minimal by-products (sodium glycolate, sodium chloride, and excess unreactive alkali) and unconverted cellulose in the produced CMC mixture. The DS value has enormous impacts on CMC’s solubility, emulsibility, thickening property, acid resistance, viscosity, stability, etc. For example, the solubility of CMC increases with the increasing value of DS [129,130]. Additionally, in emulsified drug applications, CMC acts as a better emulsifier in the 0.6–0.7 DS range than other ranges. The DS value of the CMC requires more than 0.8 for sustainable or controlled release drugs where CMC showed significant resistance against acids and salts.

Sathasivam and his co-workers (2018) [334] demonstrated a controlled release of red palm oil with its nutritional components in the targeted intestinal fluid by encapsulating them in sago biomass-derived CMC beads. Moreover, during the treatment of depleted gastric walls (ulcer), CMC is used as ulcer control media and a protective carrier of medicinal agents (leaf extracts of *Corchorus olitorius*) [335]. Furthermore, CMC is used as a binder or matrix former in pharmaceuticals. For example, anionic CMC is used as a binder with lysozyme and cur (mainly the root of turmeric). It enhances the binding capacity between lysozyme and cur during the formulation of pharmaceutical products. Additionally, it acts as a more reliable and protective carrier for the better protection of pharmacological properties against severe environments [336].

Proteins also act as a natural emulsifier and stabilizer and are used in pharmaceuticals and food products such as the Zein (ZN) protein, which is used as an emulsifier in Pickering emulsions (PEs). PEs can be used for a reduction in calorie and fat content, nutraceutical encapsulation, texture modification, and stabilize oil in water (o/w) emulsions. However, Zein encapsulation (PE) is unstable in aqueous phases and gastrointestinal conditions. Babazadeh and his fellows (2019) [337] solved this lack of Zein property (PE) by the formulation of the ZN-CMC complex. They developed a stable emulsion (W/O/W) and protected the encapsulation of nutraceuticals in gastrointestinal conditions. Moreover, this emulsion provides better particle size (89.8 ± 4.2; 83.7 ± 3.7;), nano-encapsulating parameters of hydrophobic nutraceuticals, high pH tolerability, and a more extended period of physicochemical stabilities as a result of lower release rate.

In drug delivery, the drug carrier is soundly attached to the drug. It is defined as a substrate, which maintains drug administration safety in the target area with high selectivity and effectiveness of the drug. Different cellulose and cellulose derivatives, other biocompatible compounds, etc., are widely used as drug carriers. CMC acts as the best reliable carrier for anti-cancer chemotherapy drugs. Docetaxel (DTX) is available and used as an anti-cancer chemotherapy drug. During the formulation of DTX, Tween 80 was used as a solvent to promote aqueous solubility. However, using Tween 80 has some side effects, such as severe allergic reactions and peripheral neuropathology (traumatic injuries, infections, metabolic problems, burning pain, etc.).

**Figure 8:**
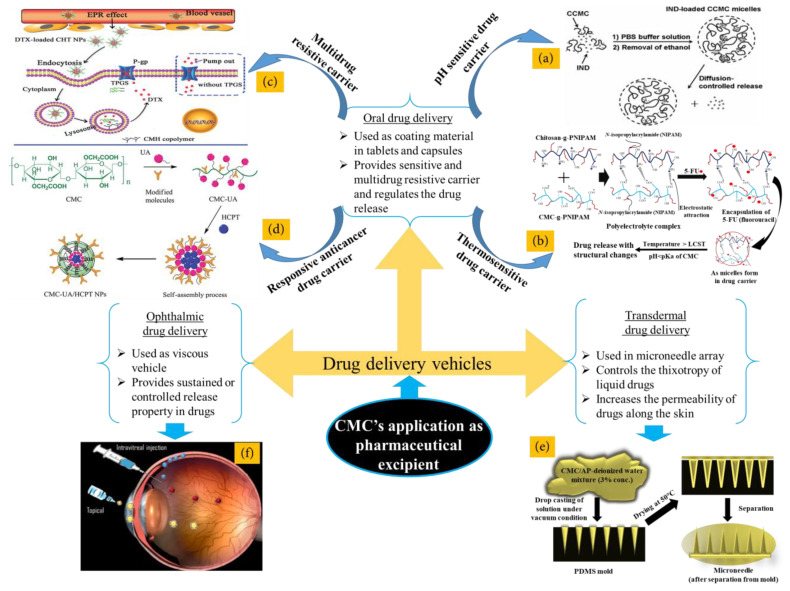
Various applications of CMC in pharmaceuticals. (**a**) Schematic representation of drug carrier function of pH-sensitive CCMC. Reproduced with permission from [341]. Copyright 2008, John Wiley and Sons; (**b**) schematic representation of temperature-sensitive drug carrier CMC-g-PNIPAM/CSg-PNIPAM (Poly-N-isopropylacrylamide) and function of the drug (5-FU) release thermosensitive carrier; (**c**) function of CMC based multidrug resistive carrier. Reproduced with permission from [338]. Copyright 2011, Royal Society of Chemistry; (**d**) function of CMC derivative in anticancer drugs [333]; (**e**) schematic representation of fabrication of CMC microneedle array for transdermal drug delivery; (**f**) the delivery path of ophthalmic drugs.

Jiang and his co-workers (2015) [338] solved these limitations of using Tween 80 with DTX by formulating nano-particle-based copolymer Na-CMC-graft-histidine and D-α-Tocopheryl polyethylene glycol 1000 succinate. Significantly, this nanocomposite helps to overcome the multidrug resistance of DTX by DTX encapsulation (Figure 8c). Similarly, we have seen other applications of CMC derivatives on anti-cancer drugs. For example, biocompatible, biodegradable graft copolymers of Na-CMC and N,N-dimethylaminoethyl methacrylate [331], the bioactive conjugated polymer of ethyl p-aminobenzoate-N-hydroxy-2,3-dihydroxybenzamide-CMC (Benzocaine-Didox-CMC conjugate) used for antitumor activity [339], aminated fumed grapheme (GO-ADH)-CMC complex drug carrier matrix (GO-CMC) used for carrying and pH-sensitive selective release of Doxorubicin hydrochloride (DOX) anticancer drug [340], chitosan-g-PNIPAM incorporated CMC-g-PNIPAM thermosensitive drug carrier used for selective release of 5-fluorouracil (anticancer drug) (Figure 8b), CMC–ursolic acid (UA) conjugate encapsulated with hydroxycamptothecin (HCPT) anti-cancer drug and formulate anti-tumor capacity based on the CMC-UA/HCPT nanoparticle [333] (Figure 8d), etc.

Furthermore, CMC conjugates are used in the oral drug delivery system for insulin. CMC is used as a mucoadhesive polymer and acts as a drug (insulin) delivery carrier for the conjugate-inhibitor form. Until now, insulin is used in the human body via subcutaneous injection (drug administration) to treat diabetes mellitus. However, this administration route is not convenient and is painful for patients. Patients need a more suitable route for insulin administration with effective delivery, high comfort, and compliance, like an oral route. Still, insulin has some limitations in the oral administration route, like low efficiency for overcoming the adsorption barrier and degraded gastrointestinal tract by enzymatic (trypsin, chymotrypsin, and elastase) degradation. As a result, insulin is not suitable for the oral route without a protective shield. Marschu et al. [342] demonstrated a new method to overcome these limitations of insulin via the oral route by formulating a protective carrier based on CMC, namely mucoadhesive polymer (CMC)-protease inhibitor conjugates such as the CMC-Bowman Birk inhibitor conjugate and CMC-Elastatinal conjugate. These conjugates provided a robust protective matrix carrier around the insulin and assured the protective and effective drug (insulin) delivery system. The most widely used gelatin-based hard capsule vehicle for oral drug supply has been replaced by a CMC-based biocomposite. In 2020, Hamdan et al. [123] developed a biocomposite-based hard capsule composed of CMC, carrageenan, and microcrystalline cellulose.

However, amphiphilic CMC derivatives or polymeric network microspheres are widely used as carriers in controlled drug release. This form of CMC showed a better vehicle property for drugs such as high solubility and biocompatibility as nano aggregates or in micellar form. For example, in hydrophobic and non-steroidal anti-inflammatory Indomethacin (IND) drugs, CMC is used as a pH-sensitive vehicle form of cholesteryl-bearing CMC derivatives (CCMC) [343] (Figure 8a). This has a better loading capacity of Indomethacin for its high attraction activity into hydrophobic drugs by the hydrophilic polysaccharide chain of CMC.

Recently, CMC has been used as an edible film-forming material in tocopherols (TC) or its derivatives. Various synthetic polymers were attached to the matrix TC to stabilize the antioxidant properties. The release of TC is not properly controllable with those matrices, and the matrix is not edible. Recently, an edible film was discovered by Martelli and his coworkers (2017) [344], composed of TC and CMC. In in vitro release, this film displayed a high efficient vehicle property for functional ingredients such as antioxidants, antimicrobial, nutrients, and flavors, etc. It is also used as a packaging material in food applications to extend the shelf-life by preventing the lipid oxidation of food (such as roasted peanuts, etc.).

In the last couple of decades to the present, CMC has been used as a viscous polymeric material for ophthalmic drug delivery systems. Sasaki and his coworkers (1999) [345] developed an emulsified Tilisolol-CMC viscose ophthalmic drug solution for the periocular injection system where CMC acted as a biocompatible emulsifier and formulated a viscous vehicle, which decreased the absorption rate by the slight leakage of drugs in the target area and improved drug delivery with the sustained-release property.

Generally, the transdermal drug delivery system (TDDS) has been widely used in protein, vaccine, and small molecule drug delivery, although it has some disadvantages. For example, the ability to pass drugs efficiently is hindered by epidermal obstruction. To mitigate these problems, Park and his coworkers (2016) [346] formulated a biocompatible and dissolvable CMC-amylopectin (CMC-AP) microneedle (Figure 8e) with a high skin permeability property to overcome the limitation of TDDS. There, the CMC primarily increased the permeability of drugs along the skin, and AP controlled the release of medications under the skin by improving the microneedle array’s dissolution property.

Unlike other review articles, here we have shown, together with the various roles of CMC in pharmaceutical applications such as binders, emulsifiers, film-forming components, reliable carriers including special property such as pH sensitive (Figure 8a) [341], thermosensitive (Figure 8b) [347], multidrug resistive (Figure 8c) [338], etc., in oral, injection, or other drug delivery systems. Additionally, their characteristics as demonstrated for use as an excipient in pharmaceuticals have been summarized in the Supplementary Materials (Table S5). Moreover, this review would be productive for researchers interested in this semi-synthetic natural polymer (CMC), especially its applications in medicine.

### 4.6. Other Applications

Up until now, CMC and its modified materials are broad terms for application purposes, so they are not appropriately confined by terms named earlier such as pharmaceutical, biomedical, tissue engineering, food, textile, and wastewater treatment. Moreover, some miscellaneous CMC uses have been proposed in various application areas including the paper industry, adhesive industry, construction industry, cosmetics products, agro products, oil industry, energy-saving purpose, etc. CMC has specific applications such as sizing, coating, adhesive, stabilizer, emulsifier, and other agents. However, in this section, the conventional applications (such as paper, sticky, or construction industry) of CMCs are designed based on CMC properties and have highlighted some advanced applications of CMCs in cosmetics or agro products, oil industry, and energy-saving purposes.

Nowadays, different paper types, including writing paper, newspaper, tissue paper, food packaging, paper or paperboard, etc., are available. To ease the mechanical strength (MS) and other properties, CMC and its derivatives are often used as a sizing or consolidation agent and coating composition. For example, during utilization as a sizing agent, CMC acts as an internal and surface sizing agent to enhance paper strength and coating quality. CMC improved the paper strength and durability by increasing hydrogen bonding between CMC and fibers with uniform distribution [348].

Moreover, CMC derivatives, namely, the modified chitosan-CMC complex was proposed (by Fatehi and his co-workers, 2009) [84,85] to enhance paper MS (dry strength), water suction ability, and a decrease in the paper damaging with their extra antimicrobial property. Similarly, Behzadi et al. (2013) proposed a new CMC-PAE (polyaminoamide-epichlorohydrin) derivative [349], which exalted the wet and dry strength of tissue paper. CMC is applied as a surface sizing agent in the newspaper that improves the surface property of paper, like ink retention on the surface and printability with lower dusting [87].

Recently, CMC and its derivatives are also applied to food packaging paper to ensure food safety from dust, grease, or microbial attack. For example, Liu and his co-workers (2014) [350] used the guanidine-grafted-dialdehyde CMC on the paper surface to make the paper more resistant to grease and enhance the antimicrobial activity. At the same time, the paper coated or grease-proofed by CMC and microbe cell membrane was destroyed by guanidine. Likewise, in waxed paper and paperboard, CMC is used as a coating material and ensures less wax penetration into the paper. For example, cellulose nanofibers (CNF)-CMC applied to the paperboard surface improve the barrier properties (high water, air and wax retention, etc.) through almost blocking the pores of the paper surface [351]. In this method, CMC provides a homogeneous coating by reducing the flocculation of micro- and nanofibers. To formulate colored papers, CMC is used with dyes or pigments on the paper surface. However, it has some limitations, such as fade-out by photochemical reaction. Later on, to improve this, CMC was used as a complex derivative form to increase the stability of the color effect. In 2008, Basta and El-Saied demonstrated the use of CMC–copper (II) complexes as a paper additive for a better coating on colored paper that provided color stability, thermal stability, and fire retardancy [86]. Generally, adhesion or binding of two different materials, adhesives, or glues is utilized. Other types of organic and inorganic bonds are applied to various adhesion applications. CMC is sometimes used as an organic polysaccharide-based adhesive for different kinds of papers and wood. The polar property of CMC provides better adhesion with cellulose-based materials (paper, wood, etc.). Commonly viscous aqueous sodium (alkali) CMC solutions, alone or with soluble starches, have been reported as forming adhesives suitable for hanging wallpaper or pasting paper products such as boxes [103]. More than that, CMC can also be used with organic or inorganic based wood adhesives for improving the binding capacity between the adhesive and wood, such as CMC/starch (organic) [89] and CMC/sodium silicate (inorganic) [352]-based wood adhesives, where CMC enhanced the adhesive performance, thermal stability, water resistance, and improved flexibility of the adhesive. Last decade, CMC was a valuable or potential binder in the Si anodic electrode for Li-ion batteries. CMC increased the stability of the solid electrolyte interphase and enhanced the cyclic performance of electrodes [90,353].

Furthermore, recently in industrial applications, CMC is used as a potential adsorbate to modify the targeted surface. CMC embeds as a binder onto the polymeric surface [354]. It is worth noticing that H. Fengyuan and his co-workers (2014) reported an essential use of CMC or CMC derivatives in the construction industry, such as CMC-sulfate on cement paste set-retarding and water-reducing agent [91]. Moreover, CMC is used as a dispersing agent in the white cement matrix while it improves the dispersion of carbon fiber in the cement matrix [92]. Cosmetic products are pretty common, and their demand has increased daily in human life for their esthetic and therapeutic effects on the skin. According to consumer demand, various cosmetics have been developed by different researchers and are available in the market, namely, skin care products, dental impression materials, etc. Along these lines, Morais et al. (2020) [355] designed a CMC/alginate-based biopolymeric 3D matrix as a vehicle of bioactive components (such as microalgae, tea tree essential oil) for application in skin care cosmetics. The 3D matrix provides a controlled release system for bioactive ingredients in derma and prevents skin disease. Based on a similar application, the CMC/hyaluronic acid-based gel or matrix was also formulated by Birsan et al. (2020) [127]. Tang et al. (2021) [356] proposed a polydopamine/CMC/polyacrylic acid-based UV protective bioactive hydrogel for skin care earlier this year.

According to Smita et al. (2010) [94], CMC is a better, comfortable, and esthetic denature adhesive (DA) in denaturing treatment in dental cases. Commonly, single CMC is often used as a naturally derived DA. Nowadays, CMC is used with Zn or Ca salt of the PMV-MA (polymethyl vinyl ether-maleic anhydride) copolymer (synthetic DA). This mixed form of DA is used to obtain better cohesive strength or covalent bonds via a carboxyl group, improving denature retention and stability. Furthermore, CMC has been used widely in the cosmetics industry as an emulsion stabilizer in creams or lotions [357]. CMC is also used with xanthan gum as an emulsion stabilizer or texturing agent in a topical cream, which formulates a better nano-emulsion for effective delivery of antioxidant coenzyme Q10 [358].

On the other hand, CMC-based hydrogels such as the CMC-PVA hydrogel is used in facial masks for improving moisture or water retention capacity [359]. Na-CMC is a dirt inhibitor agent in soap and detergent compositions [360]. For example, Agarwal and his co-workers (2012) [95] reported the application of CMC as a thickening and dirt suspending agent in liquid detergent. The following year, CMC occupied synthetic agriculture products (such as fertilizers, pesticides, insecticides, etc.) and the oil industry. In the agriculture sector, different types of fertilizers (likes nitrogen, phosphorus, potassium fertilizer, etc.) are widely applied to plants to maintain the deficiency of nutrients. However, fertilizers could create serious environmental hazards for their improper functioning after application. CMC is used as a hydrogel form in fertilizers to mitigate this problem.

Raafat et al. (2012) [96] formulated CMC/PVP (polyvinyl pyrrolidone) superabsorbent hydrogels via the gamma radiation technique for applications in nitrogen fertilizer (urea). This hydrogel could control the release rate of urea, decrease the loss rate of urea, hazard effect, and improve the proper supply of nutrients with enhanced water retention capacity on the plant. Sutradhar and his co-author (2015) [97] proposed another superabsorbent polymer (SAPs) like CMC/AAc (acrylic acid) SAPs for application for agricultural purposes. Furthermore, CMC is used with pesticides via the grafting process and acts as an emulsifier or suspending agent. For example, Chen and co-authors (2018) [361] proposed grafted CMC polymers such as CMC-g-PBA (poly butyl acrylate) or CMC-g-PS (polystyrene) or CMC-g-PMMA (polymethyl methacrylate) for application with the avermectin (AVM) pesticide and formulated AVM/grafted emulsified polymer nanoparticles. The formulated nanoparticles maintained the release of pesticides and decreased environmental pollution. CMC is used as an additive for oil drilling purposes in the oil industry. Due to the contaminating nature of fluid during use in oil drilling, Na-CMC has gained interest and is used widely as low-cost, non-contaminating, sustainable, and environmentally friendly additives in drilling fluid [98]. Additionally, CMC reduces the fluid losses and enhances the viscosity of drilling mud, and controls the thixotropic behavior. Similarly, the mixed form of CMC/xanthan gum also handled the filtrate losses of the fluid [99].

In some recent studies, CMCs have been reported as a cost-efficient binder in biomass pellets that considerably enhanced the quality, durability, and compressive strength of the biomass pellets and reduced the energy consumption extent shells [70,362]. On the other hand, due to the presence of cation-enticed carboxyl groups, CMCs can be hybridized with various metal ions such as Fe^2+^, Ca^2+^, Al^3+^, Mg^2+^, etc., via cross-linking processes to synthesize numerous aerogels with tunable physiochemical and morphological characteristics [363]. The following year, Yu et al. demonstrated a nitrogen-doped magnetic carbon aerogel from commercial CMC through multi-step approaches (involving carbonization, chemical activation, and sol-gel method) [74]. The synthesized aerogels exhibited more specific capacitance (i.e., up to 185.3 F/g) with 90.2% capacity retention after the same number of charge/discharge cycles. Furthermore, a CMC modified graphene/water nanofluid was used to store thermal energy, where CMC was used as a surfactant in nanofluid for improving the dispersion stability of a nanofluid [364]. Therefore, CMCs are also considered to be a significant component in energy storage.

## 5. Prospects

CMC and its various composites have attracted the attention of researchers during the past few decades due to their facile, low-cost synthesis process and the flexibility of its precursor materials. Many potential sources have not been explored yet in this context. Furthermore, in numerous application fields, the development of various state-of-the-art materials and more recent technologies need to be inaugurated based on CMC. Hence, several prospects that need to be addressed could inspire future research works.

Future research could focus on the following points:

(a) Utilizing various bacterial celluloses for the synthesis of high purity CMC products.
(b) Developing CMC-based nanocomposite for stimuli-sensitive control release of nutrients in the human body from food ingredients to ensure biosafety.
(c) Utilizing non-toxic and susceptible CMC for smart physiological effects (smoothing, glowing, or shining) and smart protective (UV blocking, thermo-responsive) objects in cosmetics.
(d) Most of the relevant water treatment studies are based on laboratory benchmarks. Therefore, the effectivity and efficiency of the fabricated CMC-based superabsorbent should be tested in pilot-scale studies for the removal of the targeted pollutants.
(e) Development of new biomaterials for a 3D-bioprinting process utilizing CMCs.
(f) Utilizing CMCs for in vivo bio-sensing of more organic compounds.
(g) Develop 4D bio-printing technology with more innovative and complex designs using CMC composites.

## 6. Conclusions

The flexibility and abundances of CMC precursor materials have made it more contrasting and preferable to researchers. Though the sources of CMC were confined at the initial stage of its development, a large number of their alternatives have been demonstrated by many researchers during the past several decades. Among them, corn husks were found as the most promising material that provided the maximum product yield (179.04%) with an adequate purity (i.e., 93.24%). However, in terms of the DS, the most favorable precursor material was found in the water hyacinth (i.e., 2.41). Aside from conventional sources, knitted rags provided the most promising yield of CMC (i.e., up to 1494%) with the highest DS (up to 2.84). It is worth noticing that CMCs are now mainly applied in biomedical engineering, though they are also widely used in the textiles, pharmaceuticals, and food industries as various conductive agents. Another emerging field of interest of CMCs is their efficient application in the water treatment process for the removal of other pollutants like heavy metal ions, radionuclides, dyes, etc. Though numerous hybrid materials, especially various super-absorbents based on CMC, have been reported in this context, there is still a huge prospect of developing more of them in the future. Furthermore, in energy production and storage, construction, cosmetics, oil, paper, and plastic industries, CMCs have also found a broad range of applications during the past few years. However, there are many emerging prospects in each of these fields, based on which the application of this material can be broadened further in the future.

## Data Availability

Not applicable.

## Supplementary Materials

The following are available online at https://www.mdpi.com/article/10.3390/polym13081345/s1, supplementary information contains: Figure S1: Stress-strain behavior, Figure S2: Thixotropic behavior, Figure S3: Influencing factors on CMC viscosity, Table S1: Synthesis of CMC from various plant-based precursors and their characteristics, Table S2: Synthesis of CMC from various waste materials and their characteristics, Table S3: Application of CMCs in textile, Table S4: Application of CMCs in wastewater treatment, Table S5: Application of CMCs in pharmaceuticals.
