# Supplementary material for: Recent Developments of Carboxymethyl Cellulose
Source: Polymers (Basel). 2021 Apr 20;13(8):1345. doi: 10.3390/polym13081345 (PMC8074295; doi:10.3390/polym13081345)
Supplement: Supplementary file 1 [file polymers-13-01345-s001.zip › polymers-1156615-supplementary.pdf]

Supplementary Materials

Recent Developments of Carboxymethyl Cellulose

Md. Saifur Rahman [1,*,†], Md. Saif Hasan [2,†], Ashis Sutradhar Nitai [2,†], Sunghyun Nam [3], Aneek Krishna Karmakar [2], Md. Shameem Ahsan [2], Muhammad J. A. Shiddiky [4] and Mohammad Boshir Ahmed [5,*]

[1] Department of Chemistry and Biochemistry, The University of Texas at El Paso, El Paso, TX 79968, USA
[2] Department of Applied Chemistry and Chemical Engineering, University of Rajshahi, Rajshahi 6205, Bangladesh; mshasan.ru@yahoo.com (M.S.H.); sd.ashis.ru5107@gmail.com (A.S.N.); aneek@ru.ac.bd (A.K.K.); shameem@ru.ac.bd (M.S.A.)
[3] United States Department of Agriculture, Agricultural Research Service, Southern Regional Research Center, 1100 Robert E. Lee Boulevard, New Orleans, LA 70124, USA; sunghyun.nam@usda.gov
[4] School of Environment and Science (ESC) and Queensland Micro- and Nanotechnology Centre (QMNC), Griffith University, Nathan 4111, Australia; m.shiddiky@griffith.edu.au
[5] School of Materials Science and Engineering, Gwangju Institute of Science and Technology, Gwangju 61005, Korea
* Correspondence: mrahman18@miners.utep.edu (M.S.R.); mohammad.ahmed@gist.ac.kr (M.B.A.)
† These authors contributed equally to this work.

Supplementary Information

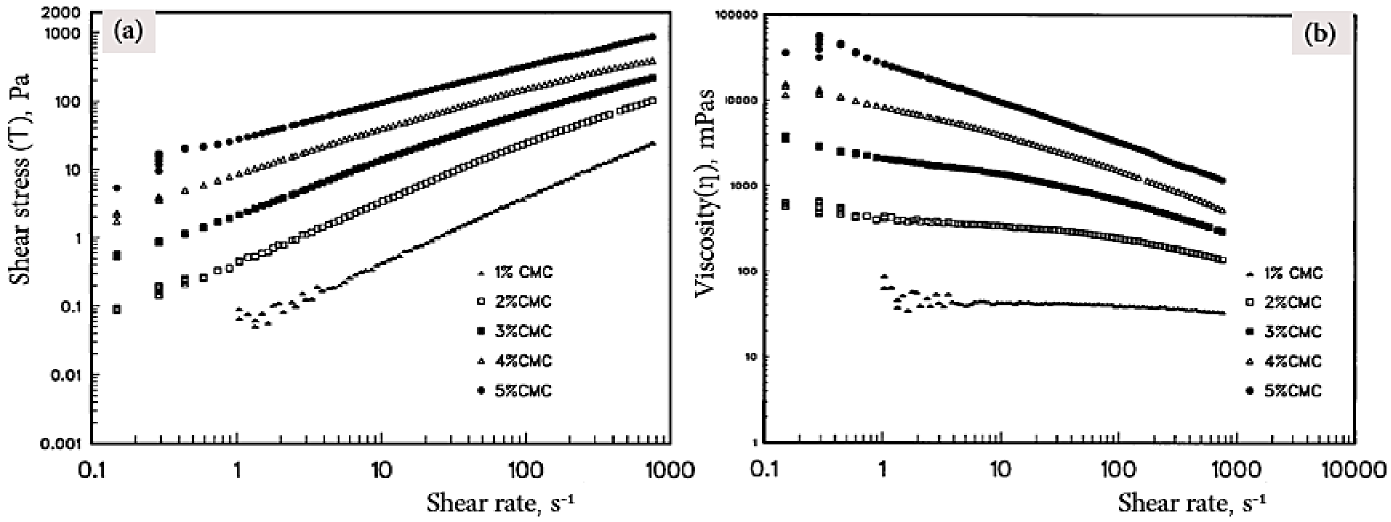

Figure S1. Stress-strain behavior. Shear stress vs. shear rate change with different concentrations (a) [1]; Viscosity change for various concentrations of solution (b) [1]. Reproduced with permission from [1]. John Wiley and Sons and Copyright Clearance Center 1998.

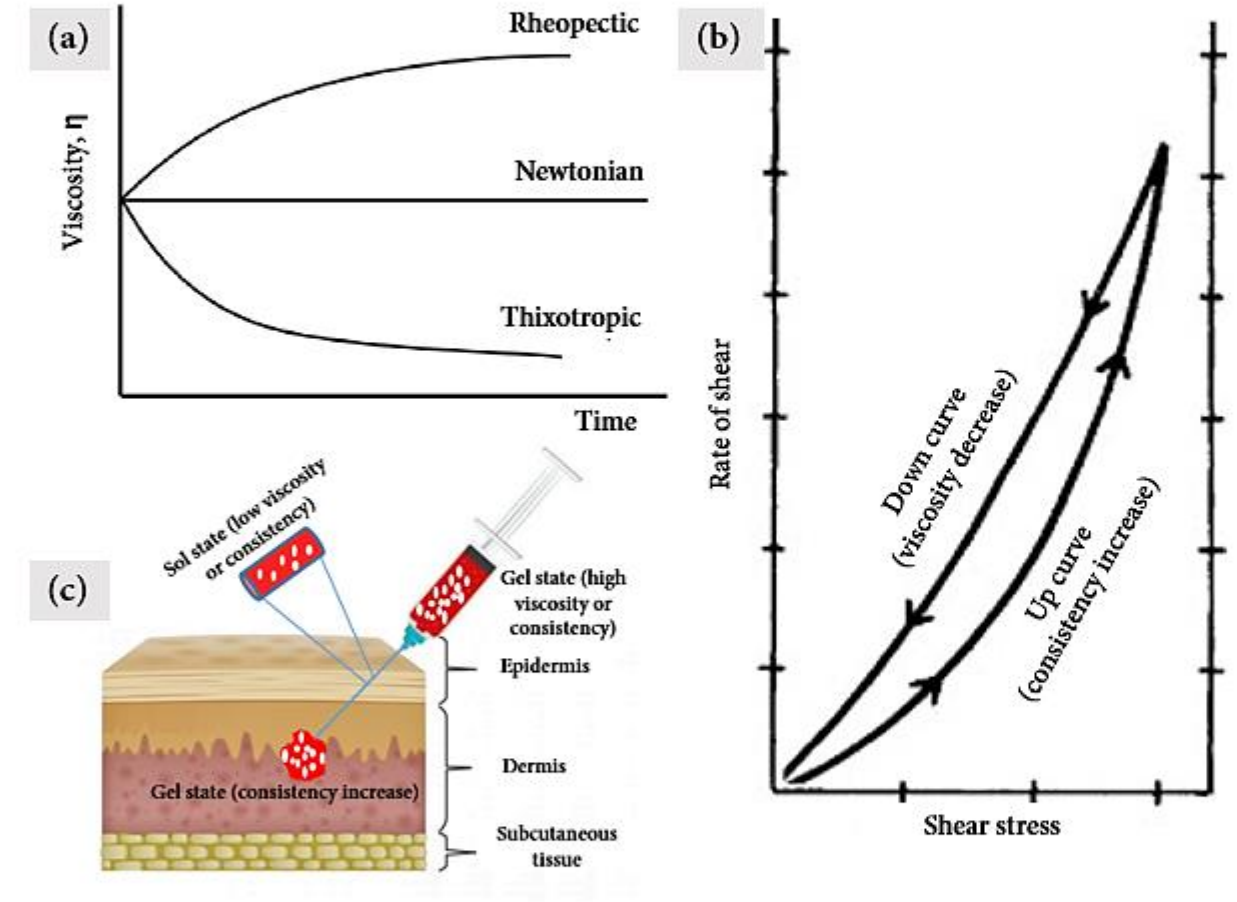

Figure S2. Thixotropic behavior. Time-dependent thixotropy vs. rheopexy, 3(a) [2]; hysteresis system of thixotropy, 3(b) [3]; mechanism of thixotropy, 3(c). Reproduced with permission from [2, 3]. Elsevier and Copyright Clearance Center; Copyright © 1991 and Copyright © 2012.

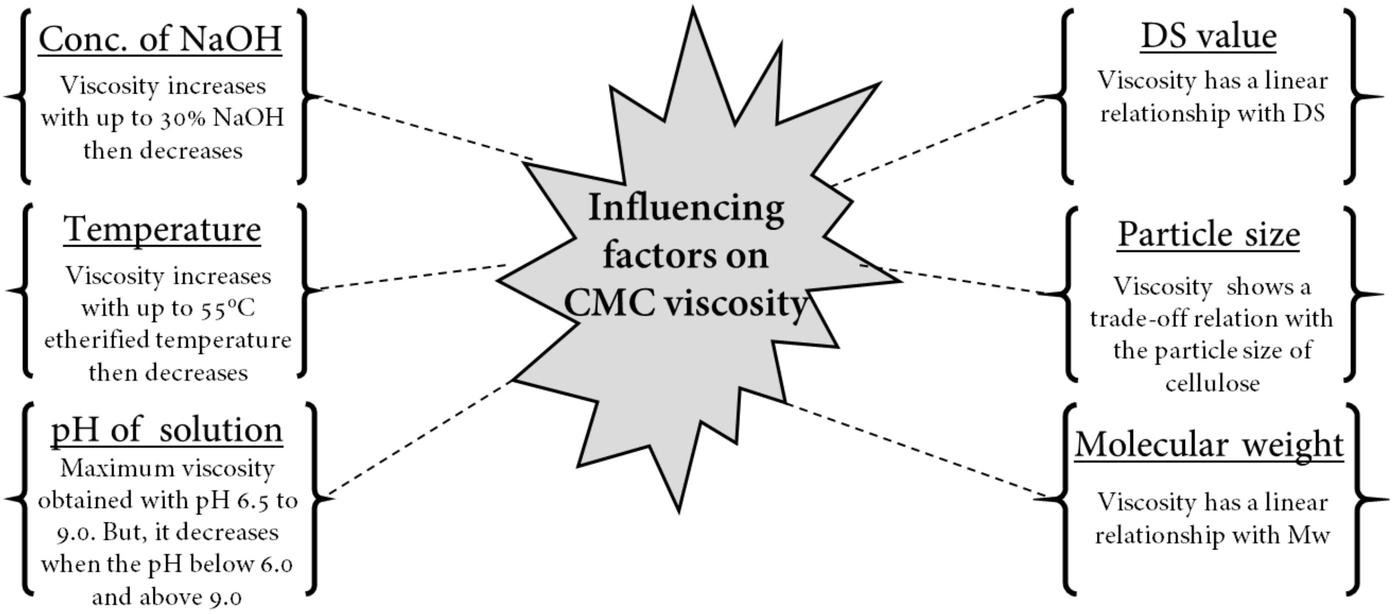

Figure S3. Influencing factors on CMC viscosity.

Table S1. Synthesis of CMC from various plant-based precursors and their characteristics.

Textile-based precursors	Cellulosic percentage and structural compositions in the precursor	Synthesis routes	Obtained degree of substitution (DS)	Obtained physiochemical properties of the synthesized CMC product	Targeted potential fields of application	Ref.
Agave lechuguilla and *Agave fourcroydes*	For *Agave lechuguilla*, Cellulose: 79.80%, Hemicellulose: 3-60%, Lignin: 15.30%, Other extracts: 2.00-4.00%. For *Agave fourcroydes*, Cellulose: 77.60%, Hemicellulose: 5.00-7.00%, Lignin: 13.10%, Other extracts: 3.60%	Extracted fibrous materials were suspended in a 2-propanol solvent and later mercerized with NaOH. This process was followed by carboxymethylation with [c]MCA at 55°C.	0.54-1.58 (obtained by HPLC analysis)	The products were obtained with high water solubility and multiple gel-forming properties.	N/A	[4]
Cotton fibers	Cellulose: 95% Non-cellulosic components including wax, pectin, protein, ash, etc.	Treated in isopropanol/water or ethanol/water medium with NaOH and monochloroacetic acid	0.3-0.4	High retention of water and antimicrobial solution	Absorbent wound dressings	[5-7]
Bamboo shaving	α-cellulose: 43.20%, Hemicellulose: 25.10%, Lignin: 15.20%.	Two-step alkylation-etherification process. (Optimum conditions- 15% NaOH and cellulose: solvent ratio = 1: 8 for alkylation, Alkylation temperature was 30˚C (70 minutes), carboxymethylation with MCA and the etherification temperature was 55˚C (90 minutes)	≥ 0.80	Degree of polymerization: 255-285, Viscosity > 260 mPa.s (experimented with 1% CMC solution)	Viscosity as well as other physiochemical properties varied with the reaction conditions (i.e., temperature, the concentration of NaOH, and reaction time). Therefore, application fields can be varied and controlled by the synthesis conditions.	[8]
Banana pseudo-stem (*Musa cavendishii* LAMBERT)	N/A	Alkylation of cellulose powder by NaOH (5-30%, the best result in 15%) at 25 °C (1hour). This process was followed by etherification with sodium-monochloroacetic acid (Na-MCA[c]) at 55 ˚C for 3 hours.	0.75	Obtained CMC purity: 98.23%, Crystallinity: 38.33%, Viscosity: 4033 cps.	N/A. (Obtained CMCs provided a moderate viscosity lower than commercial CMC products. On the other hand, as the purity of the products was less than 99.50%, it would be a potential product for various applications as a helpful technical grade CMC).	[9]
Bean hulls	N/A	Multiples pre-steps were conducted (such as pre-hydrolysis with H_2SO_4, bleaching, pulping and final hydrolysis) to obtain a microcrystalline form of CMC. The rest of the process (i.e., conventional alkylation-etherification) was optimized at the following conditions-	0.70	Moisture content: 7.70%, Molecular weight: 257.00 Appearance: white-colored Melting point: 304°C	Synthesis of antimicrobial compounds to inhibit the growth and effects of certain etiologic microbes in patients.	[10]

		Alkylation with 20% NaOH (drop-wise addition for 30 minutes, followed by 1 hour stirring at room temperature), etherification with [c]MCA (drop-wise mixing for 30 minutes followed by continuous stirring of 2 hours at 60°C)				
Cacao (*Theobroma cacao* L.) pod husk	Cellulose: 35.00%, Hemicellulose: 11.00%, Lignin: 15.00%, Mineral potassium: 3.18%, Calcium: 0.32%, Phosphorus: 0.15%	Alkylation-etherification method. (Optimum reaction conditions- 15% NaOH, 4g Na-[c]MCA, temperature 55.93°C, time= 3 hours)	0.75	Oil holding capacity: 2.12 g/g, Water holding capacity: 3.73 g/g, Viscosity: 206.10 cps, Yield: 141.60%, Lightness: 56.61	N/A	[11]
Corn cobs	α-cellulose: 34.45%, Hemicellulose: 28.23%, Klason lignin: 14.01%.	Two-step alkylation-etherification method. (Optimum yield and DS value were obtained by alkylation with 25% (w/v) NaOH treatment and etherification with 2.4 mol/AGU[a] MCA for 3 hours (at 60 °C))	1.18	Water-soluble material. Different DS values were obtained depending upon the treatment (mercerization and carboxymethylation) conditions.	N/A	[12]
Cornhusk	N/A	Alkylation-etherification process (alkylation with 30% (w/v) NaOH at 30°C, etherification with 120% (w/v) monocholoroacetic acid at 55°C, 3.5 hours)	2.41	Obtained CMC purity: >99.99%, Water retaining capacity: 5.11 g/g, Oil retaining capacity: 1.59 mg/g	As an additive in foods and pharmaceutical industries.	[13]
Cornstalk	Cellulose: 35.31%, Hemi-cellulose: 32.58%, Lignin: 18.57%, Extractives: 1.34%.	Alkylation-etherification of crude cellulose (from corn husk) with NaOH and chloroacetic acid, respectively. (75°C, 1.5 hours). To finalize the product, it was neutralized by HCl, filtered, washed with ethanol (95 vol%), and finally dried at 105 °C (3 hours).	0.57 (± 0.2%) at alkali: ether: feedstock = 4.0: 2.5: 1.0, 0.85 (± 0.40%) at alkali: ether: feedstock = 4.6: 2.8: 1.0.	Appearance: white powders, Viscosity-average molecular wt, (M_v) = 1.94 × 10^4 – 2.48 × 10^4 g/mol Water solubility: 88.6 (wt%) at alkali/CA[b]/cellulose ratio 4:2.5:1, and 95.60 (wt%) 88.60 (wt%) at alkali/CA[b]/cellulose ratio at 4.6:2.8:1. Viscosity: 8.65 (at alkali/CA[b]/cellulose ratio 4:2.5:1) and 16.34 (at alkali/CA[b]/cellulose ratio 4.6:2.8:1)	Surfactants, detergents, thickeners (in coating industries), etc.	[14]
Durian rind	N/A	Conventional alkylation-etherification process. (Alkylation was conducted by NaOH using isopropanol solvent, followed by the etherification process with MCA for 3 hours at 55°C).	0.87	Tensile strength (highest): 140.77 MPa, Rate of water-vapor transmission (highest): 220.85 g/day·m^2, The viscous property of the product was varied with temperature	Sustain releasing agent, gelling agent in pharmaceutical and biomedical applications; binding and film-forming agents in various industries; metal absorber.	[15]

				(decreased with the increase of experimental temperature).		
Eucalyptus globulus pulp	N/A	Alkylation-carboxymethylation process. Alkylation was carried out at room temperature by treating the pulp with NaOH for 1 hour, followed by the carboxymethylation process (standard slurry method). MCA was used for carboxymethylation with isopropanol solvent for 1 hour (at 60°C). Later the solid product was suspended in 80% (v/v) methanol solution, neutralized with 90% (v/v) acetic acid, washed with 70% (v/v) ethanol and pure methanol, and subsequently dried for 24 hours (60°C, vacuum).	0.87-1.32	Purity of the obtained CMC: >99.50%, Obtained viscosity: 1517 mPa.s (maximum). Linear viscoelastic range: 150-160 (maximum)	Various refined grade applications such as pharmaceuticals, foods, textiles, paints, cosmetics, adhesives, ceramics, etc.	[16]
Lantana camara L.	α-cellulose: 94.80%, Hemicellulose: 3.92%, Lignin: 0.80%, Ash: 0.48%.	Alkylation-etherification process. (Optimized experimental conditions- Alkylation by 20% (w/v) NaOH solution (3.24 mol/AGU[a]) at 25°C for 60 minutes; carboxymethylation by [c]MCA (2.05 mol/AGU[a]) at 55°C for 3.5 hours). Later the solid extract was washed with 70% (v/v) methanol solution and oven-dried at 60 °C.	1.22	Non-Newtonian pseudoplastic product, Although the obtained viscosity varied with the variation of NaOH concentration, the highly viscous product altered.	N/A	[17]
Mimosa pigra peel	N/A	Two-steps alkylation-etherification method. (Alkylation with NaOH for 1.5 hours was followed by etherifying the mercerized cellulose extent with CA[b] for 3.5 hours at 55 °C. Later the solid extracts were treated with absolute methanol, 90% (v/v) acetic acid, 70% (v/v) ethanol, and dried for 12 hours at 55 °C).	0.20-0.25	Rate of water-vapor transmission: 65.64-66.47 g/day.m^2, Permeability of water vapour: 1.51-2.28 g.m/m^2.mmHg.day, The mechanical properties varied with NaOH concentration (best mechanical properties were obtained by using 50% (w/v) NaOH solution during CMC synthesis).	Pharmaceutical, food as well as various non-food industries	[18]
Orange peel	N/A	Conventional alkylation-etherification process. (Extracted cellulose was mercerized with NaOH (90 minutes, 25 °C) and then etherified by sodium chloroacetate by continuous shredding for 360 minutes at 70	0.67	Pseudoplastic product (apparent viscosity decreased as the shear rate increased), The obtained product exhibited a time-dependent viscosity behavior, i.e.,	N/A	[19]

		°C. Later on, the obtained solid product was neutralized by 90% acetic acid, washed with 70% methanol, and finally dried at 70 °C).				
Pineapple (*Ananas comosus* L. Merryl) peel (PP-CMC)	Cellulose: 23.67%, Hemi-cellulose: 15.61%, Lignin: 6.87%, Pectin: 5.71%, Ash: 2.37%.	Modified alkylation-etherification process (alkylation with 50% (w/v) NaOH and etherification with 50% (w/v) diazomethane)	1.05 (calculated by a standard method)	Appearance: rod-like structure (diameter 60-140 μm and length 5-20 μm)	Preparation of PP-CMC-g-poly (AA-co-AM)/carclazyte superabsorbent, which was considered a potential superabsorbent hydrogel for controlled drug release, slow fertilizer release, and manufacturing of different biomaterials for numerous applications.	[20]
Posidonia wastes	N/A	Conventional two-step alkylation-etherification process. (40% NaOH solution and 17.40 g of [c]MCA was used for mercerization and carboxymethylation of each 10 g of cellulose or raw *Posidonia* waste, respectively)	0.39	The yield of CMC: 77.00%, Deionized water retention capacity and synthetic urine retention capacity were 405% and 334% better than the commercial cellulose pulps.	N/A (As the water retention capacity and the synthetic urine retention capacity of the product was far better than the commercial cellulose pulps, further applications in various medical purposes, as well as food preservation, is expected)	[21]
Oil-palm fronds	N/A	Alkylation-etherification process. (Optimized reaction conditions: 52% NaOH solution was used for mercerization (at 30°C, 1 hour), and 10.70 g of [c]MCA was used for carboxymethylation (at 50°C, 3 hours)	1.10	Highest yield of CMC: 170.10%, Purity: 97.30%, Viscosity: 685 cp (calculated for per 1% *w/v* solution of CMC),	N/A	[22]
Sago waste	Cellulose: 40-55%, Hemicellulose: 25-40%, Lignin: 15-35%	After sago pulp purification, the extracted cellulose was carboxymethylated in two-steps alkylation-etherification process. Alkylation by 30% (w/v) NaOH was conducted by dropwise addition and continue stirring for 1 hour. This procedure was followed by the etherification process with Na-[c]MCA (at 45°C, 3hours)	0.82	Appearance: light brown color with smooth surface morphology,	N/A	[23]
Sugarcane bagasse	α-cellulose: 36.70% Hemicellulose: 26.10%, Klason lignin 24.40%, Ash: 1.30%, Others: 6.10%.	Two-steps alkylation-etherification process. (Optimum conditions for maximum DS value of the product were evaluated at NaOH (conc. 28.40 g/dL), [c]MCA (1.14 g), and reaction temperature of 57.85 °C for 4.01 hours.	1.08	Crystallinity: 30.10%, Product purity: 71.60%, Yield of CMC (at optimum conditions: 181.30%)	N/A	[24]

Water hyacinth	N/A	The multi-step process was conducted to obtain pure CMC products from raw hyacinth. Dewaxing to remove pectin and wax contents, delignification (by treating with 1% $NaClO_2$ solution), removal of hemicellulose (by alkylation with 17.5% NaOH, the solvent used of the isobutanol-ethanol mixture), neutralization(with acetic acid), and finally carboxymethylation (by sodium chloroacetic acid at 60°C)	1.76	Purity: 93.24%, The highest yield of CMC: 179.04%	N/A	[25]

[a]AGU: Anhydroglucose unit, [b]CA: Chloroacetic acid, [c]MCA: Monochloroacetic acid, N/A: Not available.

Table S2. Synthesis of CMC from various waste materials and their characteristics.

Textile-based precursors	Synthesis routes	Structural composition of the precursor	Yield (%)	Obtained degree of substitution (DS)	Obtained physicochemical and morphological properties	Targeted potential fields of application	Ref.
Cotton gin waste	Cellulose was extracted from the waste precursor by delignification (with 25% NaOH, 70-80 ˚C), bleaching (by 3% NaOH and 1.5% H_2O_2), acid treatment (by H_2SO_4 and HCl), and detergent wash (70 ˚C, 1 hour). Afterward, the extracted cellulose was alkylated with NaOH for 2 hours (room temperature) and then etherified by Na-MCA[c] (3 hours).	N/A	N/A	0.87	High-quality crystalline product with moderate water immobilizing capacity.	Pharmaceuticals, cosmetics, food, ceramics, etc.	[26]
Cotton linters	Alkylation-etherification process. (Alkylation with NaOH at 30°C for 2 hours, followed by carboxymethylation with MCA (80% aqueous solution) at 55°C for 6 hours).	Composition of the linters after bleaching with H_2O_2: α-cellulose: 99.70%, Residual wax: 0.30%. (Copper number: 0.02, MB[a] adsorption value: 1.32)	N/A	0.89	Viscosity: 235 cp, Water solubility, viscosity, DS value, and other physicochemical properties of the product showed diversity upon the different concentrations of H_2O_2, which were used for bleaching of the precursor linters as a pre-treatment step.	N/A	[27]
Knitted rag	Conventional alkylation-etherification process, the temperature raised to 85°C (3 hours) for carboxymethylation.	N/A	360% (one-step carboxymethylation) to 1494% (for seven-step carboxymethylation)	0.91-2.84	Tensile strength: 0.72 MPa, Intrinsic viscosity: 0.54-0.73,	Sizing agents in yarns and fabrics.	[28]

	(Seven rounds of successive carboxymethylation were operated to investigate the change in physicochemical properties of the obtained CMC).				Molecular weight: 153,886-252,231		
Mixed office waste-paper	Conventional alkylation-etherification process. The maximum DS value was obtained at 85°C reaction temperature, 0.09 M NaOH, and 0.11 M Na-MCA[c] reagent concentrations.	N/A	150.80%	1.07	Water-soluble, non-Newtonian pseudo-plastic product (according to rheological characteristics of the obtained product in 1-3% aqueous solution).	N/A	[29]
Office-waste paper	Deinking the papers using multiple physical and chemical processes such as separation of fibers, separation of ink particles from the fibers, ink separation from the slurry, etc. These processes were followed by the refining of cellulose in multiple steps (such as pulverization, boiling with alkali solution, alcohol wash, filtering, drying, etc.) and finally, carboxymethylation of the refined cellulose in the conventional alkylation-etherification process (alkylation with NaOH for 1 hour at 35°C, etherification with Caleb for 1 hour at 70 °C).	N/A	N/A	0.81-0.82	Appearance: Canary yellow, Product purity: ≥ 90%, Viscosity: 308-316 mPa.s	In preparation of crust-dust suppressant, toothpaste as well as in paper industries.	[30]
Paper sludge	Alkylation-etherification process. Alkylation with NaOH at 40°C, etherification with sodium monochloroacetic (NaMCA) acid at 60 °C (1 hour). Optimum mass ratio: Precursor/NaOH/NaMCA = 0.90:0.80:1.15	Fiber: 86.81% (72.58% α-cellulose), Ash: 8.82%, Water content: 80% (lignin not detected).	116.60% (on a dry basis)	0.53 (average value)	The purity of CMC: 91.50%, Viscosity: 13.80 mPa.s, Crystallinity: 58.92-63.03%	In food and other packaging industries, the products can be used as water-holding agents due to their high water retention capacity.	[31]
Short cellulose fibers (SCFs) from effluents of the paper recycling industry	Slurry Process. SCFs were mixed with NaOH (20 mL alkali per 2 grams of SCFs) in the presence of isopropanol (at room temperature, 1 hour). The mercerized cellulose was etherified with MCA[c] by continuous stirring for 3 hours (55 °C) and then incubated at 30°C for 21	N/A	N/A	0.71	Water imbibing capacity: 7.0 mL/g, Degree of polymerization: 877.54.	N/A	[32]

	hours. The pure solid products were then obtained by centrifugation of the suspension and later on neutralizing with acetic acid, acetone wash, alcohol wash, and drying.						
Waste disposable paper cups	At first, the cellulose was extracted by boiling the wastepaper powders with 12% (w/v) NaOH solution, bleaching with peroxides (10% H_2O_2), washing with deionized water, and finally oven-dried for 12 hours (60°C). The extracted cellulose powders were then alkylated with NaOH at 35°C (60 minutes) and then etherified by MCA[c] (optimizing condition: 70°C, 1.5 hours, MCA dosage= 7 g).	N/A	N/A	1.21	Appearance: Rod and ribbon-like shape (diameter 25-50 μm), and thermally stable.	Potential applications in the fabrication of various flexible composites and numerous functional materials.	[33]
Waste textiles	Conventional alkylation-etherification process. (Separate cellulosic materials from the wastes were alkylated with 40% (w/v) NaOH solution. 2-propanol was used as a reaction solvent. Afterward, the alkylated intermediate product was carboxymethylated by 50% MCA[c] solution at 55 °C (3.5 hours). The final product was obtained by posterior neutralization (with 90% acetic acid), vacuum filtration, alcohol wash (with 70% ethanol, three times), drying, and finally grinding at a ball mill).	N/A	N/A	0.86	Physical appearance: Smooth powder, Water retention capacity: 541 g/g (within 60 minutes).	Fabrication of superabsorbent polymer for various water immobilizing applications. (Synthesized superabsorbent polymer from the CMC showed up to 4227 g/g water-binding capacity.	[34]

MB[a]: Methylene blue, CA[b]: Chloroacetic acid, MCA[c]: Monochloroacetic acid.

Table S3. Application of CMCs in textile.

Applied carboxymethyl cellulose or its composites	Physicochemical properties of the synthesized material	A precursor of the applied CMC	Synthesis/modification routes of the material for targeted application in the industrial field	A specific field of application	Observations	Ref.
Textile waste-derived CMC	As thickener displayed good shear thinning behavior and pseudoplastic property.	Terry towel waste (cellulosic waste)	CMC was derived from terry towel textile waste by the general alkalization-etherification process. 40% NaOH, 550C reaction temperature, and 3.5h reaction time were required.	• It is used as an excellent thickener in textile printing by providing a higher surface area for dye particles.	Showed higher K/S value (measuring color value), shear-thinning index, and lower binding length than commercial CMC. The lower binding length of dyes or fabrics provide better washability of the prints	[35]
CMC/Sodium alginate/Dextrin pre-treatment agent	Has excellent color sharpness feature with high grades.	Commercial CMC (Medium viscosity)	Composed and properly mixed three pre-treating agents like CMC, Sodium alginate, and Dextrin.	• Used in digital printing to enhance the dry or wet rubbing fastness.<br>• Used in silk fabrics for sharp printing of color.	Lightfastness, excellent coloration, or sharpness of different colors depending on the mixture of the pre-treating agent. CMC/Sodium alginate (1:1) mixture is better for black or cyan color. And CMC/Dextrin mixture better for magenta color.	[36]
CMC and starch-based hybrid; or CMC-g-polyvinyl alcohol (CMC-g-PVA)	Hybrid acts as an excellent flocculated or sizer with better adhesion of starch sizes to cotton and cotton blends. Which also has higher solubility and easily removable or washable; The grafted polymer also displayed good sizing or finishing feature.	Commercial CMC (M.W=30/1000/10000) Or, Tylose powder (commercial CMC C-30)	Starch-CMC based hybrid was formulated by respectively and individually reaction between starch, CMC, and Quat-188; Graft polymerization of Vinyl acetate in the presence of CMC followed by saponification, where acetate groups convert to hydroxyl groups to formulate CMC-g-PVA.	• In textile sizing, used as sizing or finishing agents.<br>• Used in fiber yarns for viscose staple.<br>• Used in fiber for terry towel weaving.	Show better mechanical properties than an application of CMC alone or other single sizing agents in cotton or cotton blend. Tensile strength and elongation break of CMC-g-PVA (70.20%, 10.96%) comparably better than CMC alone (67.04%, 11.12%).	[37, 38]
CMC/Acrylamide/Graphene oxide hydrogel	Provide excellent dye adsorption property.	Commercial CMC (CAS:9004-32-4)	During hydrogel formulation, CMC solution and Graphene oxide formulate their general synthesis processes, respectively. Both are mixed with Acrylamide and develop hydrogel via the free-radical polymerization process.	• Used in removing organic or hazardous dyes from textile wastewater or liquor.	The removing capacity of dye (Acid Blue-133) was higher, likes a maximum of 185.45 mg/g. And formulate hydrogel act as an environmentally friendly adsorbent.	[39]
CMC/HA or CMC/HA/Etamsylate composite based nonwoven textile	Help provide biocompatible, robust, and efficient wound healing, tissue engineering, and advanced hemostasis.	Commercial Na-CMC (DS=0.7; M.W^a= 250 g/mol)	In coagulation, the bath nonwoven was prepared by the wet spinning method.	• Used as hemostatic materials.<br>• Provide a barrier against unwanted adhesion during surgery	Provide strong, competent, and quick hemostasis ($p < 0.05$) compare to other hemostatic components. The timeline of bioresorbability is also high (up to 30 days). But, the hemostatic properties of the composite are influenced by the DS of CMC.	[40]

				• Easily blood adsorption by Na-CMC and quick interaction between blood components or fibrin with carboxy group stabilize the blood clot.		
Chitosan/CMC/glutaraldehyde emulsified microcapsule	Controlled the release of therapeutic active ingredients from cosmetic textile.	Commercial CMC (Low viscosity; M.W[a]=90000 Da)	Microcapsule formulated by crosslinking between glutaraldehyde and emulsified chitosan/CMC composed at pH 5.5.	• Used as an innovative coating of active ingredients in cosmetic textile. • CMC is used in a capsule as a charge neutralizer and stabilizer of chitosan emulsion.	Provide highly dispersed and stable layers-based microcapsule. During layer formation, the zeta potential was decreased with increasing CMC concentration.	[41]
Fumaric acid crosslinked CMC (CMCF) based silver nano-composite	Has higher antimicrobial activity with an excellent hygienic and biodegradable feature.	Commercial CMC (Low viscosity-C5678-500G)	Initially, the silver nanoparticles were formulated by the green method. Subsequently, it disperses in CMCF hydrogel. Thus, the nanocomposite is prepared.	• Generates antimicrobial activity in cotton fabric textiles.	Show excellent antimicrobial properties of CMCF with two respected concentration of silver, like $5*10^{-4}$ M and $2*10^{-3}$M silver. This reduced 30.492% and 67.377% bacteria (Staphylococcus aureus), respectively.	[42]
CMC- Polyphenols (CMC-PF) gel	Has higher antioxidant and antiradical activity with its controlled release property.	Commercial based powdered Na-CMC (Medium viscosity; M.W[a]=250000)	Polyphenols are extracted from Pomace of a blue grapevine. Following this, it is composed of CMC to formulate a CMC-PF gel.	• Provides antioxidant and antiradical activity in cotton fabric textile-based biomedical products.	In CMC-PF gel, CMC controls the release property of PF. And this release property depends on the crosslinking of CMC with PF. A higher degree of crosslinking of CMC decreases the half-time release of PF.	[43]
CMC-Hydroxyethyl Cellulose (HEC) hydrogel	Has a better pH and thermosensitive feature.	Commercial Na-CMC (M.W[a]=700 kDa, DS= 0.65–0.85)	The hydrogel is formed by the blend of CMC and HEC in Citric Acid.	• Used as a finishing agent in smart textile. • Used in textile pre-treatment for targeted stimuli-responsive purposes.	In liquid, thermosensitivity or pH sensitivity of hydrogel was mentioned by Swelling Degree (SD) of a hydrogel controlled by the concentration of Na-CMC or weight ratio of CMC/HEC. At 32^{0}C and alkaline media, the higher conc. of Na-CMC (NaCMC/HEC=3:1) in hydrogel provide higher or equivalent SD.	[44]

*M.W[a]= Molecular weight.

Table S4. Application of CMCs in wastewater treatment.

Applied CMC or its composites	Physiochemical properties of the synthesized material	A precursor of the used CMC	Synthesis/modification routes of the material for targeted wastewater treatment	Maximum removal capacity against	Optimum conditions for maximum removal of pollutants	Observations	Ref.

				the targeted pollutants			
CMC/Fe_3O_4 nanocomposite	Homogeneous magnetic nanomaterial (particle diameter~25 nm)	Mesquite tree	The conventional alkylation-etherification process to obtain CMC from the precursor. This process was followed by the co-precipitation method to synthesize the CMC/Fe_3O_4 nanocomposite	Methylene blue (MB) dye: 64 mg/g	pH= 7.0, Temperature= 25°C, Adsorbent loading= 50 mg/50 mL glass bottles	Methylene blue dyes were removed via adsorption on synthesized nanomaterial. Langmuir adsorption isotherm and pseudo-second-order reaction kinetics better described the adsorption process	[45]
Polypyrrole/ carboxymethyl cellulose (PPy/CMC) nano-hybrid material	Spherical nanomaterial (particle diameter:m 75 ±5 nm (characterized via SEM), Surface area: 48.96 m^2/g, Pore volume: 0.3886 cm^3/g.	Commercial CMC (Germany)	Co-precipitation of distilled pyrrole and the commercial CMC.	Reactive red (RR) 56 dye: 104.90 mg/g, Reactive blue (RB) 160 dye: 120.70 mg/g.	For RR56: pH= 4.0, Experimental time= 55 minutes, Adsorbent loading: 0.09g. For RB160: pH= 5.0, Experimental time= 52 minutes, Adsorbent loading: 0.09g.	Both toxic dyes were removed from the water solution via the endothermic adsorption process. The initial concentration of both of the shades was compromised at 100 mg/L for experimental work. Langmuir isotherm data better described the adsorption.	[46]
Ammonium phosphomolybdate (AMP)	Yellow microspheres	Commercial CMC (Tokyo)	AMPs were immobilized onto the commercial CMC by the chemical cross-linking process to synthesize CMC-AMP composite.	Cs^+: 64.20 mg/g	pH= 7.0, Temperature= 50 °C, Adsorbent loading= 1 g/L, Time= 24 hours.	Adsorption of the ionic pollutant was better described by Langmuir isotherm and Lagergren's first-second model, respectively. Adsorption efficiency is retained in the adsorbent up to five recycling (by	[47]

						ammonium chloride) without significant losses.	
Nitrogen-doped carbon aerogel	Quasi-spherical nanoparticles, (specific surface area 589.6 m^2/g, Pore volume: 0.27 cm^3/g)	Commercial CMC (China)	Nitrogen-doped carbon aerogels were fabricated by a group of fabrication steps, including sol-gel process, carbonization, chemical activation (using KOH), and cross-linking (ferric trichlorides were used as cross-linkers).	Methylene blue: 230.00 mg/g, Malachite green: 238.20 mg/g.	Initial conc.= 100 mg/L, Temperature= 25 °C, Time= 4 hours.	Adsorbents were more efficient for cationic dye removals than the anionic dyes.	[48]
CMC/Fe treated refined aspen wood fiber	The fibrous membrane was prepared to pass the pollutant containing experimental solution (pore measurement= 40 mesh sieve)	Commercial CMC-salt (viscosity 10-55 mPa)	Refined wood fibers were treated with an aqueous solution of CMC (1-4 wt%) and then were dried at 60 °C (oven dry). Later on, the CMC-treated fibers were soaked into $FeCl_2$ solution (2-12 wt%) and finally dried at 65 °C to obtain the adsorbent.	Phosphate: 4.30 mg/g	Initial conc. = 100 mg/L, Adsorbent dosage= 0.10 g/50 mL of phosphate solution, Shaking rate= 150 rpm, Time= 24 hours.	The fibers treated with 4%(w/w) CMC and 12% (w/w) $FeCl_2$ solution provided the maximum phosphate retention capacity.	[49]
Graphene oxide (GO)/CMC composites	Lightweight, porous monoliths. The porosity of the composites increased with the increase of GO concentrations in the structure.	Commercial CMC (China)	The CMC solution was incorporated with GO suspension by continuous stirring, ultrasonic treatment, and ice bath (10 minutes). The Solid-state of the gel mixture was obtained by bottom-to-top unidirectional freezing in a Styrofoam container and further freeze-drying (at -50 °C, 48 hours, <20 MPa vacuum pressure).	Cu^{2+}: 82.93 mg/g, Pb^{2+}: 76.70 mg/g, Cd^{2+}: 46.13 mg/g, Co^{2+}: 59.99 mg/g, Ni^{2+}: 72.04 mg/g.	Initial concentration= 40-120 mg/L, Room temperature.	Adsorption of the metal ions increased to a specific concentration of the pollutants in the experimental solution. Below or above the attention, pollutant removal decreased considerably.	[50]
CMC/polyacrylamide (PAM) composite hydrogel	Three-dimensional, highly porous, sponge-like materials. Pore diameter: 5-15 µm.	Commercial CMC (China). (DS ≥ 90%, viscosity: 800-1200 mPa.s (for 2% aqueous solution))	A free-radical polymerization method was used. In brief, three wt% CMC solution was first prepared by dissolving the commercial CMC in deionized water, previously mixed with acrylamide, N, N′-methylene-bis (acrylamide), and ammonium persulphate. The mixture was cultured for 30 minutes in a water bath (~60 °C). The subsequent lyophilization process obtained the dried hydrogel.	Cu^{2+}: 227.27 mg/g, Pb^{2+}: 312.5 mg/g, Cd^{2+}: 256.41 mg/g.	Initial pH= 5.5, Temperature= 25 °C (approx.), Shaking rate= 200 rpm, Time: 24 hours.	Adsorption behavior of the hydrogel against all of the experimental pollutants best fitted with Langmuir isotherm and pseudo-second-order reaction kinetics. In multiple pollutant adsorption, the pollutants showed selec-	[51]

						tivity according to the following order: $Cu^{2+} > Pb^{2+} > Cd^{2+}$.	
Alginate/CMC gel	Micro-structured beads (BET surface area: 2.16 m^2/g, Total pore volume: 0.0573 cm^3/g).	Commercial CMC (China). (viscosity ≥ 0.3-0.8 Pa.s (at 25 °C, 20 g/L for solution), analytical grade product).	Sodium alginate and sodium-CMC solutions (commercial) were first blended in a magnetic stirrer. The mixture was then successively cross-linked by treating $CaCl_2$ and $FeCl_3$ (1 wt% answer).	Pb^{2+}: 1.73 mg/g	Initial conc.= 5 mg/L, Temperature= 37 °C, Time= 18 hours.	Adsorption of the pollutants was best-fitted with Langmuir isotherm and pseudo-second-order reaction kinetics. Among different pH values, maximum (99.6%) adsorption was observed at the range of pH between 4 and 5.	[52]
CMC-g-poly (acrylic acid)/attapulgite hydrogel	Microporous hydrogel with 3D cross-linked polymeric networks.	Commercial CMC (China). (viscosity ≥ 300-800 mPa.s (at 25 °C, for 25 g/L solution).	CMC solution (1.03g/250 mL distilled water) was heated first and then purged with nitrogen to remove the atmospheric oxygen. Later on, ammonium persulfate (as reaction initiator), acrylic acid (as a composite component), N, N′-methylene bis(acrylamide) (as cross-linker), and attapulgite was added into it successively. The mixture was then heated for 3 hours (70 °C, nitrogen atmosphere) to complete the reaction. The final hydrogen product was obtained by subsequent drying at 70 °C.	Pb^{2+}: 941.28 mg/g	Adsorbent dosage= 0.10 g/25 mL experimental solution, Temperature= 30 °C, Shaking rate= 120 rpm, Time= 120 minutes.	Langmuir adsorption isotherm and pseudo-second-order reaction kinetics described well the adsorption process. 90% of the total adsorption occurred within 10 minutes and 30 minutes when the concentration of attapulgite in the composite was 5% and 20% (w/w), respectively.	[53]
Porous carbon aerogels (synthesized from CMC-based aerogels)	Nano-porous aerogels with interconnected 3D networks. BET surface area (after KOH-activation): 428 m^2/g, pore volume: 0.35 cm^3/g.	Commercial CMC (China).	CMC aerogels were fabricated by mixing commercially obtained CMC powders with D(+)gluconic acid-lactone, glycerol, and $Al_2(SO_4)_3$ in deionized water, respectively. The gels were then carbonized at 550 °C (raising temperature at 10 C/min heating rate) followed by subsequent	Methylene blue: 249.60 mg/g, Malachite green: 245.30 mg/g.	pH= 7.0, Initial conc.= 50 mg/L, Adsorbent dosage= 10 mg/50 mL solution, Temperature= 25 °C, Time= 12 hours.	Chemical activation with KOH significantly enhanced the adsorption capacity of the aerogels.	[54]

			physical and chemical activation. KOH-solution (15 wt%) was used for chemical activation, where physical activation was conducted at 900 °C (heating rate 5 C/min)				
Sodium carboxymethyl cellulose/Sodium styrene sulfonate (SSS) hydrogels	Grafted porous hydrogels provided rougher surface morphology as compared to the ungrafted ones. Surface roughness increased with the increase of SSS.	Commercial CMC (England). (Molecular weight~ 700,000)	20 wt% CMC paste was prepared with SSS. Later on, the pastes were centrifuged at 10,000 rpm (at least 5 hours) and subsequently irradiated with γ-ray (20-100 kGy) to obtain the final product.	Fe: 79.78 μg/g, Cr: 36.65 μg/g, Zn: 30.34 μg/g, Cu: 25.19 μg/g, Mn: 8.78 μg/g, Mo: 7.19 μg/g, Pb: 3.60 μg/g, Ni: 2.67 μg/g, U: 0.58 μg/g, Cd: 0.42 μg/g.	Optimum pH= 5.0, Temperature= 25 °C, Time= 24 hours.	Adsorption data were best fitted with Langmuir adsorption isotherm and pseudo-second-order reaction kinetics model.	[55]
Polyvinyl alcohol (PVA)/CMC hydrogel composites	The sponge-like structure with highly porous 3D networks.	Commercial CMC (Taiwan)	Hydrogels were fabricated by repetitive freeze-thaw cycles. CMC and PVA solutions were mixed at a predetermined ratio and kept at -20 °C (6 hours) for freezing. Subsequently, the gel was thawed at room temperature (1 hour) for stabilized gel formation.	Ag^{+}: 8.40 mg/g, Ni^{2+}: 6.00 mg/g, Cu^{2+}: 5.50 mg/g, Zn^{2+}: 5.30 mg/g.	Initial concentration: 100 mg/L, pH= 1.6, Temperature= 15 °C, Time= 24 hours.	Incorporating CMC in 3D networks of pure PVA hydrogel enhanced its swelling ratio (e.g., from 416% to maximum 1437%) and increased its selectivity towards ionic pollutants.	[56]
CMC-immobilized Fe_3O_4 nanocomposites	Composite nanobeads. Particle size: 7.0 nm-10.3 nm (average size: 8.7 nm), Hydrodynamic diameter: 15.7 nm, Polydispersion index 0.176, BET surface area: 198.8 m^2/g.	Commercial CMC (China). (DS value= 0.92, Viscosity= 300-800 mPa.s).	A one-step high-gravity method was used to synthesize the nanocomposites. In brief, a certain amount of $FeCl_2 .4H_2O$ and $FeCl_3 .6H_2O$ salts were added into a pre-prepared CMC solution. Another solution of NaOH was prepared at a predetermined concentration. Then, both solutions were streamed into a continuous reactor (rotating speed 1000 rpm, liquid flow at 60 L/h). The final product was obtained	Pb^{2+}: 152.00 mg/g	Adsorbent dosage: 100 mg/100 mL solution, Shaking rate= 100 rpm, Temperature= 25 °C, Time= 12 hours.	Adsorption was well fitted with pseudo-second-order reaction and Langmuir isotherm. The adsorbent showed excellent reusability even after five adsorption-desorption cycles.	[57]

			later by lyophilization (8 hours).			
Glutaraldehyde cross-linked titanium oxide/CMC nanohybrids (nano-TiO_2/glu/CMC)	Spherical homogeneous cross-linked nanoparticles. (Particle size 22.2-44.4 nm)	Commercial CMC (USA) (purity > 95%)	Nano- TiO_2, cross-linker glutaraldehyde, and CMCs were heavily mixed in a grinder for 3 hours at a predetermined ratio (i.e., 10 g of TiO_2 was mixed with 5 g of CMC by adding 10 mL of cross-linker). Later on, the cross-linked product was washed and dried for 8 hours (70 °C).	Cd^{2+}: 2440 μmol/g (at pH=7), Hg^{2+}: 1160 μmol/g (at pH=3), Pb^{2+}: 2880 μmol/g (at pH=6)	Initial conc.= 0.10 mol/L, Adsorbent dosage= 0.03 g, Time= 30 minutes.	Pollutant extraction from seawater, drinking water, and wastewater significantly varied from each other. Up to 99.6% Pb^{2+}, 88% $Cd^{2+,}$ and 83.5% Hg^{2+} were removed from the experimental seawater sample, whereas the maximum removal percentage for the respective pollutants were 100.0%, 98.3%, and 93.6% in the perspective of wastewater samples.	[58]

Table S5. Application of CMCs in pharmaceuticals.

Applied carboxymethyl cellulose or its composites	Physicochemical properties of the synthesized material	A precursor of the applied CMC	Synthesis/modification routes of the material for targeted application in the pharmaceutical field	The specific field of application	Observations	Ref.
Palm oil encapsulated CMC (PO-CMC)	Uniformly spherical beads (sphericity constant <0.5), softness, elegance, and flexibility increased by incorporating plasticizers in beads.	Sago biomass	Encapsulation of red palm oil in the obtained CMC was conducted using emulsification and subsequent ionotropic gelation (the gelling agent was aluminum chloride).	• Protection of the palm oil from oxidative stresses by encapsulation.<br>• Protection of the constituent nutrients of the oil from acid-induced degradations in the stomach.<br>• Controlled release of the nutrients in the intestinal fluids for specific nutritional or pharmaceutical actions of the constituent compounds.	Up to 22.8% (w/w), PO-CMC was produced for further application from 12.5% (w/w) emulsification of the synthesized CMC. After application in the gastrointestinal route, the beads' comparatively higher oil release and swelling behavior were demonstrated in the intestinal fluid than the stomach fluid.	[59]
A complex form of Lysozyme-CMC (Lys-CMC)	Act as the best binder and formulated protective coacervates for oral drugs, which protect the pharmacological properties of cur.	Commercial	Used phosphate buffer solution with Ly and CMC and stirring for making LY-CMC complex.	• Encapsulated of Curcumin.<br>• As binder between Lys and Cur<br>• Protective and sensitive bio activators against different physiological environments.	Better binding capacity obtained for higher DS (1.2) values of CMC. And high stable carrier for Cur.	[60]
Zein-CMC-PEG (Polyethylene glycol) Nano colloid-based water in oil in water (W/O/W) multiple emulsion system.	Act as a nano-emulsion for better nano-encapsulation of oral drug	Commercial	Used PEG-400 and Tween-80 for Zein Nano-particle production. After zein was obtained, CMC (20%) was added to the magnetic stirrer for formulating the Zein-CMC complex.	• Encapsulated and controlled the release of hydrophobic nutraceuticals/drugs.<br>• Applied against different pH conditions for the better stability of an emulsion. Such as in the stomach (1.2), intestinal (6.8), and colon (7.4).	50% Zein-CMC Nanoparticles based W/O/W emulsion provide better particle size (89.8±4.2; 83.7±3.7;) with high zeta potential (48.4±0.6; 49.2±0.4). High zeta potential provides high stability.	[61]
Graft copolymers of Na-CMC and N, N-dimethylamino ethyl methacrylate (Na-CMC-g-PDMAEMA)	Create a biocompatible, biodegradable, and conjugate-forming polymeric ampholyte composite for oral or some intravenous injection. This copolymer has stimuli-sensitivity, low toxicity, bioac-	Commercial	CMC is grafting with PDMAEMA by graft copolymerization process, where 0.5%w of potassium permanganate and 1 mL of 0.1 N HCl used as an initiator.	• Potential conjugate form with Paclitaxel (Ptx.) used as a thermo-sensitive and biocompatible carrier in cancer or tumor drug delivery.<br>• Increase the biological half-life of Ptx. by preventing its degradation under physiological condition	Lower critical solution temperature (LCST, 37-440C), stable particle size (118-133 nm) in 0.15N NaCl (aq.) solution, physiological pH range, and in vitro release in	[62]

	tivity, and antimicrobial activity properties.				acidic and neutral media at 350C. These properties of copolymer act as a suitable carrier for oral or infusion Paclitaxel Administration.
Nano-particle (MPs)based copolymer of Na-CMC-graft-histidine (CMH) and D-α-Tocopheryl polyethylene glycol 1000 succinate (TPGS), i.e., CHT-NPs	Almost similar to the Na-CMC-g-PDMAEMA copolymer properties. But CHT-NPs differ from the previous copolymer for negligible cytotoxic effect on a normal cell.	Commercial	CMC is grafting with histidine by EDC/NHS-mediated coupling reaction process, where EDC, NHS, and DMAP are used as reagents in MeCN solvent.<br><br>*EDC-- Ethyl-3-(3-dimethylaminoprop-yl)-carbodiimide HCl.<br>*NHS-- N-hydroxysuccinimide<br>*DMAP--4-dimethylaminopyridine.<br>*MeCN-- Acetonitrile	• Mitigated multidrug resistance and enhance the therapeutic effect of Docetaxel (DTX).<br>• Protected the low aqueous solubility of DTX and its allergic effects on the human body by its encapsulation.<br>• Effective drug (DTX) delivery nanocarrier against tumor or cancer cell with negligible cytotoxic effect on a normal cell.	In composite, CMH and TPGS enhanced the biocompatibility between drug (DTX) and composite at a ratio of CMH and TPGS (3:1.5). This optimum ratio help to increase the encapsulation rate of DTX in NPs. Moreover, copolymer composite maintains the smaller particle size (147.8 ± 3.14 nm) and pH-dependent drug (DTX) release behavior. Where investigated, at lower pH (4.5), DTX releases rapidly in lysosomes.	[63]
Insulin carrier dosage form of polymer-inhibitor conjugates and thiolated polymer (CMC-BBI/CMC-Ela- PCP-Cys).	Maintained drug carrier stability on oral route for insulin. Enhanced mucoadhesive properties and prolonged the resistance time of the delivery system of the drug.	Commercial	Polymer-inhibitor conjugates initially prepared by the lyophilized process. Then conjugates, thiolated polymer (PCP-Cys), and mannitol compressed with insulin	• Formulated protective carrier for insulin.<br>• Protective effect of insulin towards enzymatic attack in vitro.<br>• Provide long time stability of drug delivery and pH-sensitive release.	This system achieved over ten h for the release of a drug. The presence of PCP-Cys polymer provides more than twice mucoadhesion in vitro and enhances the stability. And the release of the drug (insulin) highly in intestinal fluid (at pH 7.1) and negligible in gastric fluid (at pH 1).	[64, 65]
Tilisolol-CMC viscose solution	Provide an emulsified viscous vehicle for controllable oph-	Commercial	Composed of Tilisolol and 3%CMC solution.	• Improved the sustain release drug and retention property in periocular tissue.	The formulated viscous vehicle showed a lower absorption rate,	[66]

	thalmic drug delivery, which improved the Tilisolol delivery into the vitreous body by avoiding side effects and drainage a drug.			• Provide the highest delivery of Tilisolol drug against aqueous humor.	higher drug retention, and best sustained-release property. And viscosity was observed at 3.7*10^5 cps.	
Transdermal CMC-Amylopectin (CMC-AP) microneedle drug delivery system	Provide a better biocompatible, easily dissolvable, and reliable system for drug delivery than others like oral or intravenous injection system, which has the first-pass metabolism of the drug along the transdermal skin.	Commercial	Microneedle prepared by a photolithography technique or laser writing process where the replica molding technique is used to fabricate a microneedle array.	• Formulated Rhodamine B drug and ascorbic acid (anti-oxidant) encapsulated microneedle array for high permeable and reliable transdermal drug delivery system. • Used on protein, vaccine, and small molecule drug delivery with controlled release property of the drug. • Applied on porcine skin to allow diffusion of ascorbic acid.	Among the various microneedles, poke and release type solid microneedles is a better reliable, soluble, and biocompatible system. Mechanical property or the drug release has been controlled by mixing CMC and PA ratios. lower dissolution property.	[67]

References

1. Ghannam, M.T.; Esmail, M.N. Rheological properties of carboxymethyl cellulose. *J. Appl. Polym. Sci.* **1997**, *64*, 289–301.
2. Dorey, R. Thick-film deposition techniques: How to make thick films—the processing techniques used to create films. *Ceramic Thick Films for MEMS and Microdevices,* 1st ed.; Elsevier, **2012**; pp. 63-83.
3. Feddersen, R.L.; Thorp, S.N. Sodium carboxymethylcellulose. *Industrial gums,* 3rd ed.; Roy, L.; Whistler, James, N. Bemiller; Elsevier, **1993**; pp. 537-578.
4. Vieira, M.; Heinze, T.; Antonio-Cruz, R.; Mendoza-Martinez, A. Cellulose derivatives from cellulosic material isolated from Agave lechuguilla and fourcroydes. *Cellulose* **2002**, *9*, 203–212.
5. Easson, M.; Villalpando, A.; Condon, B.D. Absorbent Properties of Carboxymethylated Fiber, Hydroentangled Nonwoven and Regenerated Cellulose: A Comparative Study. *J. Eng. Fibers Fabr.* **2017**, *12*, 61–69.
6. Parikh, D.; Fink, T.; Rajasekharan, K.; Sachinvala, N.; Sawhney, A.; Calamari, T.; Parikh, A.D. Antimicrobial silver/sodium carboxymethyl cotton dressings for burn wounds. *Text. Res. J.* **2005**, *75*, 134–138.
7. Parikh, D.; Sachinvala, N.; Calamari, T.; Negulescu, I. Carboxymethylated Cotton for Moist Wound Healing. *AATCC Review* **2003**, *3*, 15–19.
8. Chen, W.Q.; Lou, D.P. In *Synthesis of sodium carboxymethyl cellulose based on pretreated bamboo shaving*, Adv. Mat. Res., Trans Tech Publ: 2014; pp 169-172.
9. Adinugraha, M.P.; Marseno, D.W. Synthesis and characterization of sodium carboxymethylcellulose from cavendish banana pseudo stem (Musa cavendishii LAMBERT). *Carbohydr. Polym.* **2005**, *62*, 164–169.
10. Ibrahim, A.A.; Adel, A.M.; Abd El–Wahab, Z.H.; Al–Shemy, M.T. Utilization of carboxymethyl cellulose based on bean hulls as chelating agent. Synthesis, characterization and biological activity. *Carbohydr. Polym.* **2011**, *83*, 94–115.
11. Hutomo, G.S.; Marseno, D.W.; Anggrahini, S. Synthesis and characterization of sodium carboxymethylcellulose from pod husk of Cacao (Theobroma cacao L.). *Afr. J. Food Sci.* **2012**, *6*, 180–185.
12. Singh, R.K.; Singh, A.K. Optimization of reaction conditions for preparing carboxymethyl cellulose from corn cobic agricultural waste. *Waste Biomass Valor* **2013**, *4*, 129–137.
13. Mondal, M.I.H.; Yeasmin, M.S.; Rahman, M.S. Preparation of food grade carboxymethyl cellulose from corn husk agrowaste. *Int. J. Biol. Macromol.* **2015**, *79*, 144–150.
14. Shui, T.; Feng, S.; Chen, G.; Li, A.; Yuan, Z.; Shui, H.; Kuboki, T.; Xu, C. Synthesis of sodium carboxymethyl cellulose using bleached crude cellulose fractionated from cornstalk. *Biomass Bioenergy* **2017**, *105*, 51–58.
15. Rachtanapun, P.; Luangkamin, S.; Tanprasert, K.; Suriyatem, R. Carboxymethyl cellulose film from durian rind. *LWT—Food Sci Technol* **2012**, *48*, 52–58.
16. Dapía, S.; Tovar, C.A.; Santos, V.; Parajó, J.C. Rheological behaviour of carboxymethylcellulose manufactured from TCF-bleached milox pulps. *Food Hydrocoll.* **2005**, *19*, 313–320.

17. Mondal, M.I.H.; Yeasmin, M.S.; Rahman, M.S.; Sayeed, M.A. Synthesis and characterization of high-purity food grade carboxymethyl celluloses from different parts of maize waste. *Cellulose and cellulose derivatives,* Mondal, M.I.H.; Nova Science publisher, New York, USA, **2015**; pp. 227.
18. Rachtanapun, P.; Rattanapanone, N. Synthesis and characterization of carboxymethyl cellulose powder and films from Mimosa pigra. *J. Appl. Polym. Sci.* **2011**, *122*, 3218–3226.
19. Yaşar, F.; Toğrul, H.; Arslan, N. Flow properties of cellulose and carboxymethyl cellulose from orange peel. *J. Food Eng.* **2007**, *81*, 187–199.
20. Dai, H.; Huang, H. Enhanced swelling and responsive properties of pineapple peel carboxymethyl cellulose-g-poly (acrylic acid-co-acrylamide) superabsorbent hydrogel by the introduction of carclazyte. *J. Agric. Food Chem.* **2017**, *65*, 565–574.
21. Ramzi, K.; Ikhlass, F. Study of liquids absorption and retention capacities of new cellulosic materials and sodium cellulose carboxylmethylate prepared from posidonia. *Fibers Polym.* **2010**, *11*, 593–597.
22. Tasaso, P. Optimization of reaction conditions for synthesis of carboxymethyl cellulose from oil palm fronds. *Int. J. Chem. Eng. Appl.* **2015**, *6*, 101.
23. Pushpamalar, V.; Langford, S.J.; Ahmad, M.; Lim, Y.Y. Optimization of reaction conditions for preparing carboxymethyl cellulose from sago waste. *Carbohydr. Polym.* **2006**, *64*, 312–318.
24. Golbaghi, L.; Khamforoush, M.; Hatami, T. Carboxymethyl cellulose production from sugarcane bagasse with steam explosion pulping: Experimental, modeling, and optimization. *Carbohydr. Polym.* **2017**, *174*, 780–788.
25. Saputra, A.H.; Qadhayna, L.; Pitaloka, A.B. Synthesis and characterization of carboxymethyl cellulose (CMC) from water hyacinth using ethanol-isobutyl alcohol mixture as the solvents. *Int. J. Chem. Eng. Appl.* **2014**, *5*, 36–40.
26. Haleem, N.; Arshad, M.; Shahid, M.; Tahir, M.A. Synthesis of carboxymethyl cellulose from waste of cotton ginning industry. *Carbohydr. Polym.* **2014**, *113*, 249–255.
27. Jahan, I.A.; Sultana, F.; Islam, M.N.; Hossain, M.A.; Abedin, J. Studies on indigenous cotton linters for preparation of carboxymethyl cellulose. *Bangladesh J Sci Ind Res* **2007**, *42*, 29–36.
28. Fakrul Alam, A.; Mondal, M.I.H. Utilization of cellulosic wastes in textile and garment industries. I. Synthesis and grafting characterization of carboxymethyl cellulose from knitted rag. *J. Appl. Polym. Sci.* **2013**, *128*, 1206–1212.
29. Joshi, G.; Naithani, S.; Varshney, V.; Bisht, S.S.; Rana, V.; Gupta, P. Synthesis and characterization of carboxymethyl cellulose from office waste paper: A greener approach towards waste management. *Waste Manage. (Oxford)* **2015**, *38*, 33–40.
30. Li, S.; Zhou, G.; Liu, Z.; Wang, N.; Wei, Z.; Liu, W. Synthesis and performance characteristics of a new ecofriendly crust-dust suppressant extracted from waste paper for surface mines. *J. Clean. Prod.* **2020**, *258*, 120620.
31. He, X.; Wu, S.; Fu, D.; Ni, J. Preparation of sodium carboxymethyl cellulose from paper sludge. *J. Chem. Technol. Biotechnol.* **2009**, *84*, 427–434.
32. Mastrantonio, G.; Battaioto, L.; Jones, C.; Coustet, M.; Chandi, H.; Yamul, D.K. Chemical conversion of paper industry effluents into carboxymethylcellulose. *Process Saf. Environ. Prot.* **2015**, *94*, 315–321.
33. Chen, J.; Li, H.; Fang, C.; Cheng, Y.; Tan, T.; Han, H. Synthesis and structure of carboxymethylcellulose with a high degree of substitution derived from waste disposable paper cups. *Carbohydr. Polym.* **2020**, *237*, 116040.
34. Bidgoli, H.; Zamani, A.; Jeihanipour, A.; Taherzadeh, M. Preparation of carboxymethyl cellulose superabsorbents from waste textiles. *Fibers Polym.* **2014**, *15*, 431–436.
35. Sheikh, J.; Bramhecha, I.; Teli, M. Recycling of terry towel (cellulosic) waste into carboxymethyl cellulose (CMC) for textile printing. *Fibers Polym.* **2015**, *16*, 1113–1118.
36. Jeong, D.-S.; Chun, T.-I. Color Fastness of Digital Textile Printing on Silk Fabrics-The effect of the mixed pre-treatment agent. *Fashion Text. Res. J.* **2013**, *15*, 808–814.
37. Hebeish, A.; Higazy, A.; El-Shafei, A.; Sharaf, S. Synthesis of carboxymethyl cellulose (CMC) and starch-based hybrids and their applications in flocculation and sizing. *Carbohydr. Polym.* **2010**, *79*, 60–69.
38. Mohamed, Z.E.-S.; Amr, A.; Knittel, D.; Schollmeyer, E. Synthesis and application of new sizing and finishing additives based on carboxymethyl cellulose. *Carbohydr. Polym.* **2010**, *81*, 769–774.
39. Varaprasad, K.; Jayaramudu, T.; Sadiku, E.R. Removal of dye by carboxymethyl cellulose, acrylamide and graphene oxide via a free radical polymerization process. *Carbohydr. Polym.* **2017**, *164*, 186–194.
40. Suchý, P.; Paprskářová, A.; Chalupová, M.; Marholdová, L.; Nešporová, K.; Klusáková, J.; Kuzmínová, G.; Hendrych, M.; Velebný, V. Composite Hemostatic Nonwoven Textiles Based on Hyaluronic Acid, Cellulose, and Etamsylate. *Mater. Corros.* **2020**, *13*, 1627.
41. Roy, J.; Ferri, A.; Salaün, F.; Giraud, S.; Chen, G.; Jinping, G. Chitosan-carboxymethylcellulose based microcapsules formulation for controlled release of active ingredients from cosmeto textile. *IOP Conf. Ser: Mater. Sci. Eng.* **2017**, *254*, 072020.
42. Bozaci, E.; Akar, E.; Ozdogan, E.; Demir, A.; Altinisik, A.; Seki, Y. Application of carboxymethylcellulose hydrogel based silver nanocomposites on cotton fabrics for antibacterial property. *Carbohydr. Polym.* **2015**, *134*, 128–135.
43. Krizova, H.; Wiener, J. Development of carboxymethyl cellulose/polyphenols gels for textile applications. *Autex Res. J.* **2013**, *13*, 33–36.
44. Gorgieva, S.; Kokol, V. Synthesis and application of new temperature-responsive hydrogels based on carboxymethyl and hydroxyethyl cellulose derivatives for the functional finishing of cotton knitwear. *Carbohydr. Polym.* **2011**, *85*, 664–673.
45. Salama, A.; Etri, S.; Mohamed, S.A.; El-Sakhawy, M. Carboxymethyl cellulose prepared from mesquite tree: New source for promising nanocomposite materials. *Carbohydr. Polym.* **2018**, *189*, 138–144.

46. Tanzifi, M.; Yaraki, M.T.; Beiramzadeh, Z.; Saremi, L.H.; Najafifard, M.; Moradi, H.; Mansouri, M.; Karami, M.; Bazgir, H. Carboxymethyl cellulose improved adsorption capacity of polypyrrole/CMC composite nanoparticles for removal of reactive dyes: Experimental optimization and DFT calculation. *Chemosphere* **2020**, *255*, 127052.
47. Zhang, N.; Chen, S.; Hu, J.; Shi, J.; Guo, Y.; Deng, T. Robust and recyclable sodium carboxymethyl cellulose–ammonium phosphomolybdate composites for cesium removal from wastewater. *RSC adv.* **2020**, *10*, 6139–6145.
48. Yu, M.; Han, Y.; Li, J.; Wang, L. Magnetic N-doped carbon aerogel from sodium carboxymethyl cellulose/collagen composite aerogel for dye adsorption and electrochemical supercapacitor. *Int. J. Biol. Macromol.* **2018**, *115*, 185–193.
49. Eberhardt, T.L.; Min, S.-H.; Han, J.S. Phosphate removal by refined aspen wood fiber treated with carboxymethyl cellulose and ferrous chloride. *Bioresour. Technol.* **2006**, *97*, 2371–2376.
50. Zhang, Y.; Liu, Y.; Wang, X.; Sun, Z.; Ma, J.; Wu, T.; Xing, F.; Gao, J. Porous graphene oxide/carboxymethyl cellulose monoliths, with high metal ion adsorption. *Carbohydr. Polym.* **2014**, *101*, 392–400.
51. Godiya, C.B.; Cheng, X.; Li, D.; Chen, Z.; Lu, X. Carboxymethyl cellulose/polyacrylamide composite hydrogel for cascaded treatment/reuse of heavy metal ions in wastewater. *J. Hazard. Mater.* **2019**, *364*, 28–38.
52. Ren, H.; Gao, Z.; Wu, D.; Jiang, J.; Sun, Y.; Luo, C. Efficient Pb (II) removal using sodium alginate–carboxymethyl cellulose gel beads: Preparation, characterization, and adsorption mechanism. *Carbohydr. Polym.* **2016**, *137*, 402–409.
53. Liu, Y.; Wang, W.; Wang, A. Adsorption of lead ions from aqueous solution by using carboxymethyl cellulose-g-poly (acrylic acid)/attapulgite hydrogel composites. *Desalination* **2010**, *259*, 258–264.
54. Yu, M.; Li, J.; Wang, L. KOH-activated carbon aerogels derived from sodium carboxymethyl cellulose for high-performance supercapacitors and dye adsorption. *Chem. Eng. J.* **2017**, *310*, 300–306.
55. Tran, T.H.; Okabe, H.; Hidaka, Y.; Hara, K. Removal of metal ions from aqueous solutions using carboxymethyl cellulose/sodium styrene sulfonate gels prepared by radiation grafting. *Carbohydr. Polym.* **2017**, *157*, 335–343.
56. Wang, L.-Y.; Wang, M.-J. Removal of heavy metal ions by poly (vinyl alcohol) and carboxymethyl cellulose composite hydrogels prepared by a freeze–thaw method. *ACS Sustain. Chem. Eng.* **2016**, *4*, 2830–2837.
57. Fan, H.; Ma, X.; Zhou, S.; Huang, J.; Liu, Y.; Liu, Y. Highly efficient removal of heavy metal ions by carboxymethyl cellulose-immobilized Fe3O4 nanoparticles prepared via high-gravity technology. *Carbohydr. Polym.* **2019**, *213*, 39–49.
58. Mahmoud, M.E.; Abdou, A.E.; Sobhy, M.E.; Fekry, N.A. Solid–solid crosslinking of carboxymethyl cellulose nanolayer on titanium oxide nanoparticles as a novel biocomposite for efficient removal of toxic heavy metals from water. *Int. J. Biol. Macromol.* **2017**, *105*, 1269–1278.
59. Sathasivam, T.; Muniyandy, S.; Chuah, L.H.; Janarthanan, P. Encapsulation of red palm oil in carboxymethyl sago cellulose beads by emulsification and vibration technology: Physicochemical characterization and in vitro digestion. *J. Food Eng.* **2018**, *231*, 10–21.
60. Li, Z.; Wang, Y.; Pei, Y.; Xiong, W.; Zhang, C.; Xu, W.; Liu, S.; Li, B. Curcumin encapsulated in the complex of lysozyme/carboxymethylcellulose and implications for the antioxidant activity of curcumin. *Food Res. Int.* **2015**, *75*, 98–105.
61. Babazadeh, A.; Tabibiazar, M.; Hamishehkar, H.; Shi, B. Zein-CMC-PEG multiple nanocolloidal systems as a novel approach for nutra-pharmaceutical applications. *Adv. Pharm. Bull.* **2019**, *9*, 262–270.
62. Sorokin, A.V.; Kuznetsov, V.A.; Lavlinskaya, M.S. Synthesis of graft copolymers of carboxymethyl cellulose and N, N-dimethylaminoethyl methacrylate and their study as Paclitaxel carriers. *Polym. Bull.* **2020**, 1-18.
63. Jiang, W.; Yang, L.; Qiu, L.; Xu, J.; Yang, X.; Wang, J.; Zhou, H.; Wang, D. Multifunctional hybrid nanoparticles based on sodium carboxymethylcellulose-graft-histidine and TPGS for enhanced effect of docetaxel. *RSC adv.* **2015**, *5*, 53835–53845.
64. Marschütz, M.K.; Bernkop-Schnürch, A. Oral peptide drug delivery: Polymer–inhibitor conjugates protecting insulin from enzymatic degradation in vitro. *Biomaterials* **2000**, *21*, 1499–1507.
65. Marschütz, M.K.; Caliceti, P.; Bernkop-Schnürch, A. Design and in vivo evaluation of an oral delivery system for insulin. *Pharm. Res.* **2000**, *17*, 1468–1474.
66. Sasaki, H.; Kashiwagi, S.; Mukai, T.; NISHIDA, K.; NAKAMURA, J.; NAKASHIMA, M.; ICHIKAWA, M. Topical Delivery System of Ophthalimic Drugs by Periocular Injection with Viscous Solution. *Biol. Pharm. Bull.* **1999**, *22*, 961–965.
67. Park, Y.-H.; Ha, S.K.; Choi, I.; Kim, K.S.; Park, J.; Choi, N.; Kim, B.; Sung, J.H. Fabrication of degradable carboxymethyl cellulose (CMC) microneedle with laser writing and replica molding process for enhancement of transdermal drug delivery. Biotechnol. Bioprocess Eng. 2016, 21, 110–118.
